# Novel Adhesive Nanocarriers Based on Mussel-Inspired Polyglycerols for the Application onto Mucosal Tissues

**DOI:** 10.3390/pharmaceutics14050940

**Published:** 2022-04-26

**Authors:** Keerthana Rajes, Peer Nölte, Cynthia V. Yapto, Kerstin Danker, Henrik Dommisch, Rainer Haag

**Affiliations:** 1 Institute of Chemistry and Biochemistry, Freie Universität Berlin, Takustr. 3, 14195 Berlin, Germany; keerthana.rajes@fu-berlin.de (K.R.); peer.noelte@fu-berlin.de (P.N.); 2 Institute of Biochemistry, Charité-Universitätsmedizin Berlin, Corporate Member of Freie Universität Berlin, Humboldt Universität zu Berlin, and Berlin Institute of Health, 10117 Berlin, Germany; cynthia.yapto@charite.de (C.V.Y.); kerstin.danker@charite.de (K.D.); 3 Department of Periodontology, Oral Medicine and Oral Surgery, Charité—Universitätsmedizin Berlin, Corporate Member of Freie Universität Berlin, Humboldt-Universität zu Berlin, and Berlin Institute of Health, 14197 Berlin, Germany; henrik.dommisch@charite.de

**Keywords:** CMS nanocarriers, adhesion, catechol, oral mucosa, drug delivery

## Abstract

A synthetic route for adhesive core-multishell (CMS) nanocarriers for application to the oral mucosa was established using mussel-inspired catechol moieties. The three CMS nanocarriers with 8%, 13%, and 20% catechol functionalization were evaluated for loading capacity using Nile red, showing an overall loading of 1 wt%. The ability of Nile red loaded and functionalized nanocarriers to bind to a moist mucosal surface was tested in two complementary adhesion assays under static and dynamic conditions using monolayers of differentiated gingival keratinocytes. Adhesion properties of functionalized nanocarriers were compared to the adhesion of the non-functionalized nanocarrier. In both assays, the CMS nanocarrier functionalized with 8% catechol exhibited the strongest adhesion compared to its catechol-free counterpart and the CMS nanocarriers functionalized with 13% and 20% catechol.

## 1. Introduction

The mucosal tissues of the oral cavity can undergo a range of inflammatory diseases such as gingivitis and periodontitis [1]. Periodontitis in particular is a widely encountered issue, with about half the population suffering from mild to moderate forms of this disease, and 10 to 12% suffering from its severe and rapidly progressing forms [2,3]. Topical drug formulations are a feasible treatment method for the oral mucosa due to these tissues’ high accessibility, robust blood supply, and fast regeneration [1].

However, treatment of mucosal tissues still faces an array of challenges, due mostly to the highly dynamic oral environment and its coverage by saliva, which decrease treatment agents’ ability to adhere to the oral mucosa. Saliva also dilutes drug formulations, further decreasing their bioavailability, and periodontal diseases increase the flow rate of gingival crevicular fluid [4]. Therefore, drugs and drug carriers intended for mucosal application must be formulated with adhesive properties to counteract these inherent limits to adhesion in the oral mucosal environment.

Multivalency is used to enhance the binding of mucoadhesive nanoparticles: these particles can achieve adhesion by electrostatic interactions, van der Waals interactions, hydrophobicity, and entangled chains, as well as by hydrogen bonding to mucosal tissue [5,6].

Catechol groups are reported in the literature for their adhesive properties. 3,4-dihydroxy-*L*-phenylalanine (DOPA) is an amino acid with catechol groups found in its side chains that play a substantial role in the adhesion of mussels underwater onto a variety of wet surfaces [7,8,9]. Inspired by the adhesive nature and catechol functionality of DOPA, a variety of catechol-based adhesive polymers have been developed, such as the DOPA-functionalized poly(ethylene glycol) (PEG) chains reported by Catron et al. in 2006 [10]. These functionalized polymers also include the nanogels and micelles based on 5-methyl-5-allyloxycarbonyl-1,3-dioxan-2-one (MAC) and PEG polymers that were functionalized with DOPA as described by Zhang et al. [11]. Lee et al. reported the use of surface-adherent polydopamine films for surface coating, opening new research avenues for mussel-inspired polymers [12].

For steric reasons, the nanoparticles used for drug encapsulation should not be larger than the mucosal mesh-pore size range of 10–200 nm [13]. Larger structures such as the nanogels and micellar systems mentioned above, with a size range of 100–200 nm, should therefore be considered carefully [10,11]. For example, Schimpel et al. described 500 nm particles that were able to diffuse through human mucus and reasoned that the viscoelasticity of this system plays a role in penetration properties [14]. Two important limitations of the reported micelles are that their structure is based on self-assembly and that their formation is dependent on critical micellar concentration (CMC). At lower concentrations, micellar structures readily disintegrate, releasing their cargo too early—a major drawback in the saliva-dominated oral cavity. By contrast, core-multishell (CMS) structures, defined by a stable structure with covalent bonds, do not depend on weak non-covalent interactions for structural integrity. With a dendritic core functionalized with amphiphilic linear chains that form double shells, CMS structures can host a range of hard-to-solubilize drugs while maintaining these treatment agents’ solubility. Their heavily modifiable structures make them adaptable to a range of drugs and applications. For instance, modifying the hydrophobicity of the shells can enable them to host a range of hydrophobic and hydrophilic drugs. In addition, changes to the shells’ internal cavities, size, and drug- or environment-specific fine-tuning, to name a few possible adjustments, can afford highly target-specific drug delivery [15].

An early dendritic catechol-based polymer was described by Wei et al., who functionalized hyperbranched polyglycerol (hPG) with catechol groups that were used to crosslink and form an adhesive surface coating [16,17]. The resulting coating was reported to have stronger adhesive properties than its polydopamine derivatives. Various mussel-inspired dendritic polymers have been reported for functional surface coatings, including coatings with anti-fouling properties [18,19,20,21,22,23,24,25,26,27,28,29]. The potential application of catechol-functionalized polymers for disease treatment has also been studied: catechol-functionalized polymers showed increased adhesion and thereby increased the bioavailability of the encapsulated drugs [7,30,31]. Meanwhile, our previous work included the intensive study of nonfunctionalized CMS nanocarriers for application in the oral mucosa [1,32]. These studies revealed that the release of the hydrophobic compound dexamethasone from the nanocarrier is superior compared to the release from a conventional cream, which is normally used for the treatment of inflammatory conditions in the oral cavity.

With existing literature strongly suggesting the promise of catechol functionalization in constructing drug carriers, our study aimed to build on this work (and on our own CMS nanocarrier research) by synthesizing catechol-functionalized CMS nanocarriers with different degrees of catechol and by comparing their adhesive properties with nonfunctionalized CMS nanocarriers.

This work describes the synthesis and characterization of catechol-functionalized CMS nanocarriers and demonstrates their adhesive properties. Drug loading was tested, and stability tests were performed. Drug release in CMS nanocarriers is reported through a variety of release mechanisms including diffusion and enzymatic degradation of the carrier [33,34]. To demonstrate the nanocarriers’ adhesive nature, adhesion tests were carried out on moist cell surfaces of differentiated oral epithelial cells. It is hypothesized that catechol functionalization of the CMS nanocarriers improves adhesion to the oral mucosa as compared to the non-functionalized CMS-C_0_ nanocarrier.

## 2. Materials and Methods

### 2.1. Materials

Laboratory glassware was oven-dried before use. Reactions were heated with a stirring bar and a heater with temperature control.

PEG (M_w_ = 400 Da) and mPEG (M_w_ = 400 Da) were purchased from TCI (TCI Deutschland GmbH, Eschborn, Germany); dihydrocaffeic acid **4** was purchased from Sigma Aldrich (St. Louis, MO, USA), pentadecane dioic acid and PCl_3_ from ABCR (abcr GmbH—Karlsruhe, Deutschland), and Dimethylaminopyridine (DMAP) from Acros Organics (Fair Lawn, NJ, USA). hPG-OH (10 kDa) was synthesized according to Roller et al., while the non-adhesive CMS nanocarrier was synthesized according to Unbehauen et al. [34,35]. Merck TLC Silica gel 60 F254 was used for thin-layer chromatography. Products were visualized using iodine on silica. Solvents were purchased as chemical reactant grade unless stated otherwise. Anhydrous acetone was purchased as ultra-dry solvents from Acros Organics (Fair Lawn, NJ, USA).

### 2.2. Methods

#### 2.2.1. Nuclear Magnetic Resonance (NMR)

^1^H-NMR spectra were acquired by JEOL ECP 500 (JEOL (Germany) GmbH, Freising, Germany) and Bruker AVANCE 500 (Bruker Corporation, Billerica, MA, USA) at room temperature, and the data were processed by MestReNova Version 14.1.1. The chemical shifts *δ* are given in parts per million (ppm) in relation to tetramethylsilane using the residual solvent peak as a reference (MeOH, *δ* ^1^H = 3.31 ppm, ^13^C = 77.16 ppm). Coupling constants *J* are reported in Hertz (Hz). The multiplicity of peaks is quoted as *s* = singlet, *d* = doublet, *t* = triplet, *q* = quartet, and *m* = multiplet. NMR spectra of the synthesized compounds can be found in the supporting information (Figures S1–S7).

#### 2.2.2. Carrier Synthesis

Catechol **5**

Dihydrocaffeic acid **4** (6.00 g, 32.93 mmol, 1.0 equiv.) was dissolved in dry acetone (50 mL). PCl_3_ (1.0 equiv.) was added at 0 °C and stirred for 2 h. After removal of the solvent under reduced pressure, the residue was redissolved in diethyl ether, and the supernatant was collected. Removal of the solvent yielded a white solid (5.46 g, 24.57 mmol, 75%).

^1^H NMR (500 MHz, MeOD) δ = 6.63 (m, 3H), 2.82 (t, *J* = 7.6, 2H), 2.56 (t, *J* = 7.6, 2H), 1.63 (s, 6H).

PEG-Catechol **6**

PEG (1.61 g, 7.24 mmol, 1.0 equiv.) was stirred with catechol **5** (2.90 g, 7.24 mmol, 1.0 equiv.) in bulk at 10 mbar and 70 °C bath temperature for 2 h. The reaction mixture was purified using column chromatography (R_f_ = 0.6 in 10% MeOH/dichloromethane (DCM)) to obtain a colorless liquid (1.65 g, 2.73 mmol, 38%).

^1^H NMR (500 MHz, MeOD) δ = 6.61 (m, 3H), 4.19 (m, 2H), 3.63 (m, 34H), 2.81 (m, 2H), 2.58 (m, 2H), 1.61 (d, *J* = 1.8, 6H).

Double shell **2**

Pentadecane dioic acid (14.46 g, 53.09 mmol, 3.0 equiv.) was melted at 130 °C. mPEG (7.08 g, 17.70 mmol, 1.0 equiv.) was slowly added, and the bulk mixture was stirred at high vacuum (HV) at 130 °C bath temperature for 5 h. The bulk mixture was dispersed in DCM (100 mL), and the precipitate was filtered off. The filtrate was further concentrated and stored in the fridge overnight. The formed precipitate was filtered off. The solvent of the filtrate was removed under reduced pressure to afford a white wax-like solid (9.14 g, 14.00 mmol, 82%).

^1^H NMR (500 MHz, MeOD) δ = 4.21 (m, 2H), 3.63 (m, 32H), 3.54 (m, 2H), 3.36 (s, 3H), 2.33 (t, *J* = 7.4, 3H), 2.28 (t, *J* = 7.4, 3H), 1.61 (q, *J* = 7.4, 4H), 1.31 (m, 18H).

Double shell **3**

PEG-Catechol **6** (1.66 g, 2.75 mmol, 1.0 equiv.) was stirred with pentadecane dioic acid (2.25 g, 8.26 mmol, 3.0 equiv.) in bulk at HV at 130 °C bath temperature for 4 h. The reaction mixture was purified using column chromatography (MeOH/DCM) to obtain a colorless liquid (1.26 g, 1.35 mmol, 49%).

^1^H NMR (500 MHz, MeOD) δ = 6.62 (m, 3H), 4.20 (m, 4H), 3.64 (m, 32H), 2.83 (t, *J* = 7.5, 2H), 2.60 (t, *J* = 7.6, 2H), 2.33 (td, *J* = 7.4, 2.9, 2H), 2.27 (t, *J* = 7.4, 2H), 1.62 (m, 10H), 1.31 (m, 18H).

CMS Nanocarriers

General procedure: Double shell **2** and double shell **3** were dissolved in dry dimethylformamide (DMF) (35 mL). Dicyclohexylcarbodiimide (DCC) (1.1 equiv.) and DMAP (1.0 equiv.) were added under cooling at 0 °C. The reaction was then stirred for 15 min. Afterwards, the mixture was added to hPG-OH in DMF (1.0 equiv. in 3 mL) and stirred for 20 h. The murky mixture was concentrated, filtered, and dialyzed for 3 d. Removal of the solvent yielded the product. GPC spectra of the synthesized compounds can be found in the supporting information (Figures S8–S10).

*CMS-C_0.2_.* General procedure using double shell **2** (2.41 g, 3.69 mmol, 0.8 equiv.), double shell **3** (0.90 g, 0.2 equiv.), DCC (0.70 g, 5.28 mmol, 1.1 equiv.), DMAP (0.06 g, 0.53 mmol, 0.11 equiv.), hPG (0.35 g, 4.80 mmol, 1.0 equiv. OH).

^1^H NMR (500 MHz, MeOD) δ = 6.62 (d, *J* = 5.5, aromatic-H), 4.20 (m), 4.05 (tt, *J* = 12.1, 3.6, aliphatic-H), 3.63 (m, PEG backbone), 3.36 (s, 2H, PEG backbone), 2.83 (t, *J* = 7.6, cat-H), 2.60 (t, *J* = 7.5, cat-H), 2.34 (m), 1.84 (m), 1.62 (m), 1.38 (m).

*CMS-C_0.13_.* General procedure using double shell **2** (2.57 g, 4.07 mmol, 0.85 equiv.), double shell **3** (0.67 g, 0.71 mmol, 0.15 equiv.), DCC (0.67 g, 5.27 mmol, 1.1 equiv.), DMAP (0.06 g, 0.53 mmol, 0.11 equiv.), hPG (0.35 g, 4.79 mmol, 1.0 equiv. OH).

^1^H NMR (500 MHz, Methanol-d_4_) δ = 6.62 (d, *J* = 5.2, aromatic-H), 4.21 (m), 4.05 (tt, *J* = 12.1, 3.6), 3.63 (m, PEG backbone), 3.35 (s, PEG backbone), 2.83 (t, *J* = 7.6, cat-H), 2.60 (t, *J* = 7.5, cat-H), 2.34 (tt, *J* = 7.2, 3.7), 1.84 (m), 1.62 (m), 1.38 (m).

*CMS-C_0.08_.* General procedure using double shell **2** (2.73 g, 4.31 mmol, 0.9 equiv.), double shell **3** (0.45 g, 0.48 mmol, 0.1 equiv.), DCC (0.67 g, 5.27 mmol, 1.1 equiv.), DMAP (0.06 g, 0.53 mmol, 0.11 equiv.), hPG (0.35 g, 4.79 mmol, 1.0 equiv. OH).

^1^H NMR (500 MHz, MeOD) δ = 6.62 (d, *J* = 4.3, aromatic-H), 4.21 (m), 4.05 (tt, *J* = 12.0, 3.6), 3.63 (m, PEG backbone), 3.35 (s, PEG backbone), 2.83 (t, *J* = 7.5, cat-H), 2.60 (t, *J* = 7.5, cat-H), 2.34 (td, *J* = 7.4, 2.8), 1.85 (m), 1.62 (m), 1.31 (m).

#### 2.2.3. Dynamic Light Scattering (DLS)

DLS measurements were performed using a Malvern Zetasizer Nano instrument (Malvern Instruments Ltd., Worcestershire, UK) equipped with a He-Ne laser (633 nm). To measure the hydrodynamic diameter (D_H_), the CMS nanocarriers were dissolved in MilliQ water, and 100 µL of the solution were added to a disposable Plastibrand micro cuvette (Brand GmbH + Co KG, Wertheim, Germany) with a round aperture. The measurements were performed at 25 °C after equilibrating the system at this temperature for 30 s. A detector angle of 173° (backscattering mode) was used. The size distribution was determined with the Zetasizer DLS software (Malvern Instruments Ltd., Worcestershire, UK).

#### 2.2.4. ζ-Potential

Measurements were performed using a Malvern Zetasizer Nano instrument (Malvern Instruments Ltd., Worcestershire, UK). One mg/mL of the CMS carrier solution in a 5 mM PB buffer was added into a folded capillary cell (Malvern Analytics). The measurements were performed at 25 °C after calibrating the system at this temperature for 30 s. The ζ-potential was determined with the Zetasizer DLS software (Malvern Instruments Ltd., Worcestershire, UK).

#### 2.2.5. Ultraviolet and Visible Spectroscopy (UV Vis)

Measurements were performed using the Agilent Cary 8454 UV-Visible spectrophotometer. Half-micro quartz cuvettes with a 4 × 10 mm light path from Suprasil from Hellma analytics (Hellma GmbH & Co. KG, Müllheim, Germany) or Spectrosil (Carl Roth, Karlsruhe, Germany) were used. The data were collected using the UV-Vis ChemStation Software. The drug loading of the nanocarriers was determined by freeze-drying 50 µL of the drug-carrier solution and dissolving it in acetonitrile before UV Vis spectroscopy. The concentration was determined using a calibration curve.

#### 2.2.6. Deprotection of the CMS Nanocarrier

The required amount of the CMS carrier was dissolved in methanol and stirred at 80 °C for 3 h to initiate deprotection of the catechol groups.

#### 2.2.7. Cryo Transmission Electron Microscopy (Cryo-TEM) Measurements

Perforated carbon film-covered microscopical 200 mesh grids (R1/4 batch of Quantifoil, MicroTools GmbH, Jena, Germany) were cleaned with chloroform and hydrophilized by 60 s glow discharging at 10 mA in a Safematic CCU-010 device (safematic GmbH, Zizers, Switzerland). Subsequently, 4 μL aliquots of the sample solution were applied to the grids. The samples were vitrified by automatic blotting and plunge freezing with a FEI Vitrobot Mark IV (Thermo Fisher Scientific Inc., Waltham, MA, USA) using liquid ethane as cryogen.

The vitrified specimens were transferred to the autoloader of a FEI TALOS ARCTICA electron microscope (Thermo Fisher Scientific Inc., Waltham, MA, USA). This microscope is equipped with a high-brightness field-emission gun (XFEG) operated at an acceleration voltage of 200 kV. Micrographs were acquired on a FEI Falcon 3 direct electron detector (Thermo Fisher Scientific Inc., Waltham, MA, USA) using a 100 μm objective aperture.

#### 2.2.8. Nile Red (NR) Encapsulation

A total of 5 mg/mL of the deprotected CMS carrier solution in MilliQ water were stirred with 50 wt% NR for 22 h. The solution was then filtered using a 0.45 µm filter (regenerated cellulose). The NR concentration was determined using UV VIS spectroscopy.

#### 2.2.9. Cell Culture

hTERT-immortalized human gingival keratinocytes (OKG4; kindly provided by Susan Gibbs, Amsterdam) were used for the experiments [36,37]. To test the adhesion of nanocarriers under static conditions, 2.5 × 10^4^ cells were seeded in each well of 96-well polystyrol microplates (black, Greiner Bio-One, Kremsmünster, Austria) and cultured in DermaLife K medium (CellSystems, Troisdorf, Germany) containing 60 µM Ca^2+^ (LifeLine^®^ Cell Technology, Frederick, MD, USA), supplemented with 1% penicillin/streptomycin (PAN Biotech, Aidenbach, Germany). For the adhesion test under dynamic conditions, OKG4 cells were seeded at 3.5 × 10^4^ cells/well on a collagen IV-coated coverslip (13 mm diameter; Karl Hecht GmbH & Co. KG, Sondheim vor der Rhön, Germany) placed in a 24-well-plate (Corning, NY, USA). At 80–90% confluency, cell differentiation was induced by increasing the Ca^2+^ level in the medium to 1.4 mM.

#### 2.2.10. Static Adhesion Assay

The binding of NR-loaded CMS nanocarriers was tested by incubating a monolayer of differentiated gingival keratinocytes, OKG4, with the nanocarrier for different time points. Before incubation with nanocarriers, the medium was removed from the cells, and 20 µL of the new cell culture medium containing 1.4 mM Ca^2+^ at 37 °C were then added for 10 min. Subsequently, 50 µg/mL of the nanocarrier (diluted in cell culture medium) were added to the cells, which were then incubated at 37 °C for 5 min, 15 min, and 30 min. The unbound nanocarrier was removed, and cells were washed twice with PBS (Sigma-Aldrich, St. Louis, MO, USA). The fluorescence intensity of NR-loaded CMS nanocarriers and cell autofluorescence were read out using the GloMax Discover Microplate reader (Promega, Madison, WI, USA) with an excitation wavelength of 520 nm and emission wavelength in the range of 580–640 nm. As a loading control, the fluorescence of 20 µL of each NR-loaded nanocarrier was determined. Cells incubated without the nanocarrier served as a negative control. From the values obtained, the arithmetic mean was taken, and standard deviations were calculated. Three independent experiments were performed in duplicate.

#### 2.2.11. Dynamic Adhesion Assay

Additionally, a dynamic adhesion test was developed and performed. For this test, the coverslip was placed on a petri dish (35 mm diameter; Corning, NY, USA) with an incline angle of 35°. Cells were washed once with PBS, and then the respective NR-loaded CMS nanocarrier (50 µg/mL) was passed along the cell monolayer one, five, or ten times at a constant rate of flow. To remove the unbound nanocarrier, the cell monolayer was washed with PBS and subsequently fixed with 4% paraformaldehyde in PBS (Sigma-Aldrich, St. Louis, MO, USA). Cells incubated with cell culture medium alone served as a negative control.

For fluorescence analysis and subsequent quantification, the nanocarrier-treated cell monolayers were counterstained. Nuclei were visualized with Hoechst 33,342 dye (H0, Invitrogen, Waltham, MA, USA), and cell membranes were stained with AlexaFluor 488-conjugated Wheat Germ Agglutinin (WGA) (Invitrogen, Waltham, MA, USA). The signal of NR-loaded CMS and CMS catechol nanocarriers that bound to the cell monolayer was visualized using a LSM700MAT confocal microscope (Zeiss, Wetzlar, Germany). The histogram setting of the red channel was set in the same setting using ZEN (Blue Edition—Version 3.4, Zeiss, Wetzlar, Germany). The acquired images were further processed using ImageJ (Version 1.53).

All experiments were performed in triplicate. In each experiment, four to six images of each treatment were acquired using identical settings of exposure and processing.

To quantify NR fluorescence, mean grey values of defined areas were recorded at four different locations in each image. To exclude the background signal, the mean grey value of the negative control was subtracted. From the values obtained, the arithmetic mean was taken, and standard deviations were calculated. Images of each technical replicate considered for the quantification are provided as supplemental material (Figures S16–S24).

#### 2.2.12. Statistical Analysis

For statistical analysis, GraphPad Prism 9 (La Jolla, CA, USA) was used. Here, the normal distribution of mean values in each treatment was tested using the D’Agostino-Pearson normality test and verified with the Shapiro–Wilk normality test. For normally distributed values, the one-way ANOVA test corrected by Tukey’s multiple comparison test was performed to determine the significant difference between all treatments. Otherwise, the significant difference was tested using Dunn’s multiple comparisons test.

## 3. Results and Discussion

### 3.1. Rational Design

**Figure 1:**
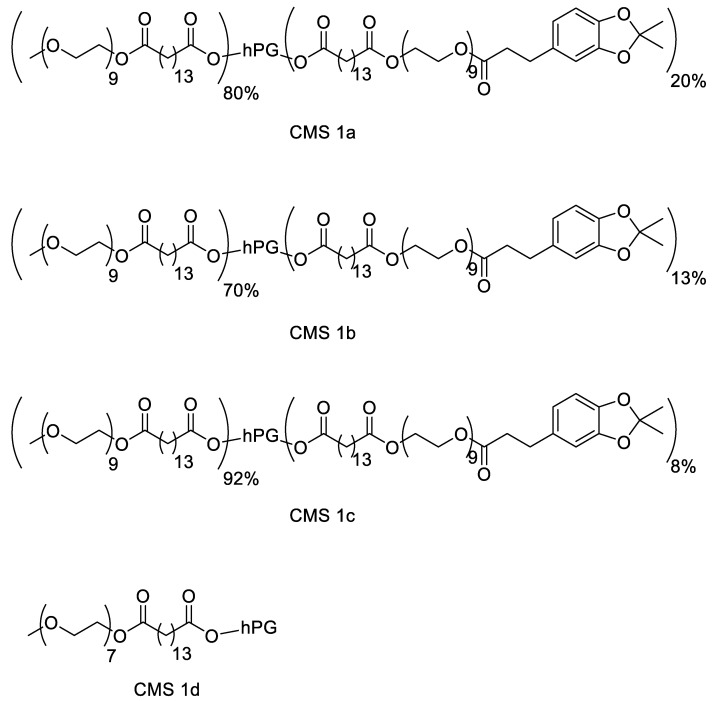
Structure of the catechol-functionalized core-multishell (CMS) nanocarriers and the catechol-free CMS.

The motivation for synthesizing a biodegradable nanocarrier system with adhesive properties for better adhesion to and penetration into mucus is based on biomimicry of mussel adhesion on wet surfaces. A dendritic core-multishell structure was chosen using hPG as the core due to its high biocompatibility and easy functionalization. The amphiphilic double shell consisted of a hydrophobic alkyl chain as an inner shell, and an outer shell consisted of a PEG chain. PEG not only aids solubility in aqueous media due to its hydrophilic nature but is also reported for its mucus-penetrating properties [38,39]. To enhance the mucus-adhering and mucus-penetrating properties of the carrier, the surface was functionalized using catechol groups in mimicry of mussel foot proteins [16,40,41]. To obtain a biodegradable polymer, ester bonds were chosen for the connection of all fractions. To investigate the effect of the catechol functionalization on mucosal drug delivery, three variations of the nanocarrier were synthesized with 8, 13, and 20% catechol functionalization. To prevent aggregation due to the catechol group, higher degrees of functionalization were avoided. Additionally, the catechol moieties were synthesized in their protected forms to prolong their storage stability. A similar CMS nanocarrier without catechol moieties, as reported by Unbehauen et al., was used for comparison [34]. The structures of the CMS nanocarriers are shown in Figure 1.

### 3.2. Material Synthesis

**Scheme 1:**
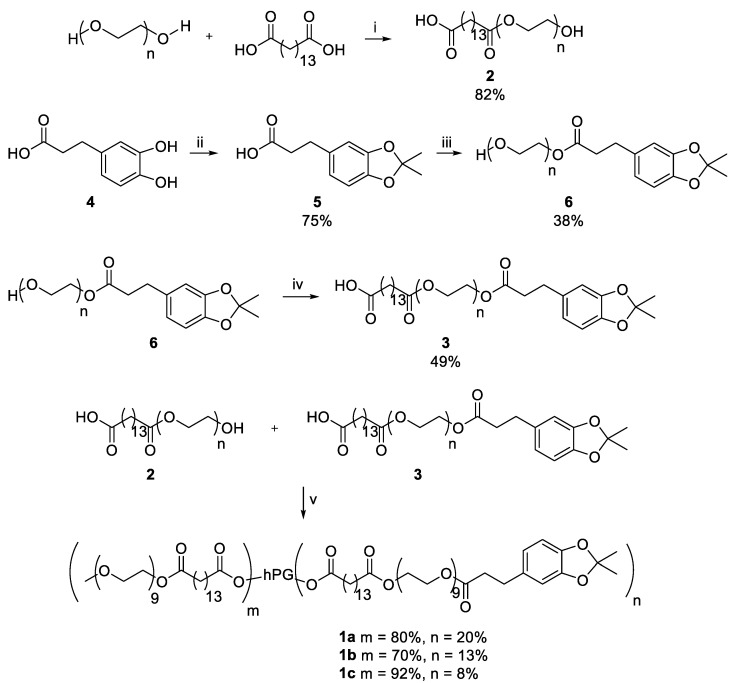
Synthesis of the adhesive CMS nanocarriers. The following conditions were used: (i) 130 °C, high vacuum (HV), 5 h; (ii) 0 °C, acetone, 2 h; (iii) 70 °C, 10 mbar, 2 h; (iv) 130 °C, HV, 4 h; (v) (1) Dicyclohexylcarbodiimide, Dimethylaminopyridine, dimethylformamide (DMF), 0 °C, 30 min; (2) hyperbranched polyglycerol, DMF, r.t., o.n.

The CMS nanocarriers were synthesized by coupling amphiphilic double shells onto hPG. Double shell **2** was synthesized by an ester bond formation of mPEG and pentadecane dioic acid with a yield of 82%. For double shell **3**, catechol **4** was first protected using acetone at 0 °C, yielding the protected catechol **5** with a yield of 75%. It was then attached to PEG, creating an ester bond and yielding PEG-Catechol **6** with a yield of 38%. Conversion of pentadecane dioic acid with PEG-Catechol **6** led to the double shell **3** with a yield of 49%. The double shells **2** and **3** were then attached to hPG using DCC and DMAP to obtain the nanocarrier CMS-C_0.2_ with 20% catechol coupling, nanocarrier CMS-C_0.13_ with a catechol coupling of 13%, and nanocarrier CMS-C_0.08_ with 8% catechol coupling. An overview of the synthesis route is given in Scheme 1.

### 3.3. Size and Morphology

The synthetic process aimed for 20%, 15%, and 10% catechol-functionalized CMS nanocarriers. To determine the final degree of functionalization (DF), the ^1^H NMR spectra were analyzed. All three carriers were expected to have a theoretical molar mass of around 102–106 kDa. The nanocarrier CMS-C_0.2_ yielded a 20% catechol functionalization, with an overall functionalization of the hPG with double shells of 100% based on NMR analysis. Nanocarrier CMS-C_0.13_ gave an actual DF of 13% catechol instead of 15%, and a total functionalization with double shells of 83%, yielding a weight of 86 kDa as compared to the 104 kDa theoretical weight. The nanocarrier CMS-C_0.08_ yielded an 8% functionalization, with an overall 100% functionalization of the hPG with double shells. The size was determined using DLS measurements. All three carriers displayed a hydrodynamic diameter of 10 nm in water with a PDI of 0.7 to 1 indicating a broad size distribution. All three carriers display a ζ-potential ranging between three to five which correlates to their neutral charge. Table 1 gives an overview of the functionalization and size of the CMS nanocarriers. The DF calculation is shown in the supporting information.

### 3.4. Deprotection and Stability Test

**Figure 2:**
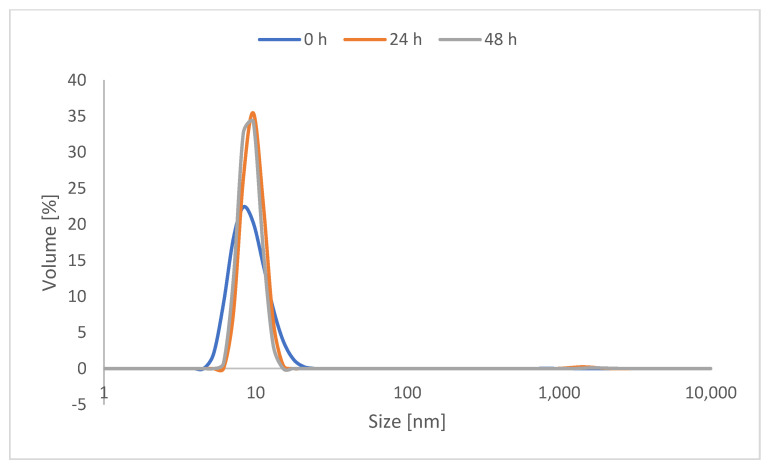
DLS measurement of the volumetric hydrodynamic diameter of CMS-C_0.2_ right after deprotection, after 24 h, and after 48 h.

As catechol is known in the literature for inter-crosslinking, protected catechol was used for the synthesis of the CMS nanocarriers to prolong their storage stability. Before use, the required amount of carrier was deprotected by stirring at 80 °C for 3 h. The solvent was then removed under reduced pressure to be exchanged with an aqueous solvent. To observe the stability of the deprotected CMS carrier, a 48-h study was performed using the deprotected version of the most highly catechol-functionalized nanocarrier, with 20% catechol functionalization. The dynamic light scattering of 5 mg/mL of the deprotected CMS-C_0.2_ in MilliQ water was then measured after 24 h and 48 h. As evident in Figure 2, notable aggregation was not evident within 48 h through measuring the hydrodynamic diameter by volume.

It must be noted that catechol-functionalized CMS nanocarriers as well as CMS nanocarriers in general are prone to some degree of aggregation in water [34]. The DLS measurements also show aggregation when looking at the protected carrier system (see Figures S11 and S12). However, aggregations with respect to the hydrodynamic diameter by intensity are multiplied and do not display the actual ratio. These are taken into consideration when looking at the volume ratio and therefore display the actual ratio better.

Drug carrier formulations also showed no aggregations despite all CMS nanocarriers being stored for several days. It must be noted that deprotected catechol is subject to oxidation and can exist in its oxidized quinone form.

**Figure 3:**
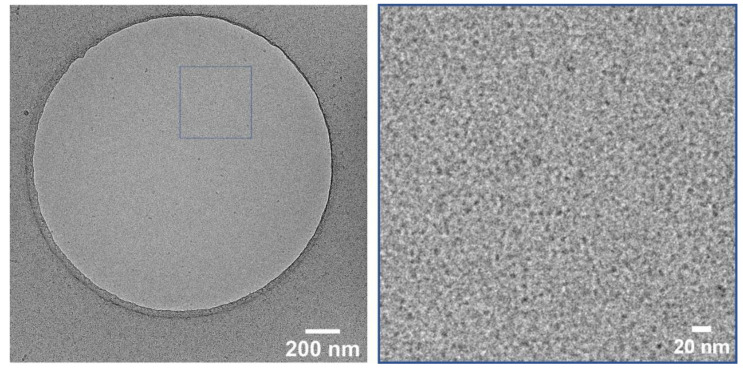
Cryo-TEM image of CMS-C_0_ at 28k magnification (**left**) and enlarged section of the marked area (**right**) taken after deprotection.

For a better understanding of the stability, cryo-TEM measurements were performed using the deprotected CMS-C_0.08_ carrier. Figure 3 depicts the state of the carrier solution after deprotection where no aggregations are visible, and carriers with a size of roughly 10 nm are visible. Cryo-TEM measurements taken after 2 days of deprotection show the same characteristics further supporting the stability of the deprotected carrier solutions.

### 3.5. Drug Loading

To investigate their drug encapsulation properties, the CMS nanocarriers were encapsulated with NR as a model hydrophobic drug. For this, 50 wt% of NR was stirred with a 5 mg/mL carrier solution in water for 22 h. To remove the excess non-encapsulated drug, the solution was filtered using a 0.45 µm syringe filter. The supernatant then only contained the encapsulated drug analogue particles as well as the amount of drug that naturally dissolves in water due to the formation of aggregates in case of excess NR. As the amount of naturally dissolvable NR is negligible, 1% is essentially the amount of encapsulated NR. A drug concentration of 1 wt% was found in all NR-loaded CMS nanocarriers, which correlates with the drug loading of nanocarriers with similar structures [42,43].

### 3.6. Adhesive Properties

Current antiseptic and/or anti-inflammatory formulations suffer from several drawbacks when considered for topical application. Their effectiveness is constrained by limited bioavailability, duration of application, and modes of application [44]. Many drug formulations show poor adhesion to oral mucosal surfaces, particularly due to the dynamic flushing effect in oral mucosa, demonstrating the need for new formulations with strong adhesive properties.

Several studies show catechol-functionalized polymers to be promising bioadhesive polymers that may have potential as mucoadhesive delivery systems for topical application in the oral cavity [7,30,45]. CMS nanocarriers are carrier systems that could be used for drug delivery in the oral mucosa. We demonstrated that they adhere to and penetrate the oral mucosal tissues. They also released the glucocorticoid dexamethasone, which is normally used to treat certain inflammatory conditions in the oral cavity better than a conventional topical cream [1,32]. All these studies prompted us to investigate further whether mussel-inspired catechol functionalization can enhance the adhesive properties of the CMS nanocarrier, further improving its potential as a mucoadhesive delivery system for topical application in the oral cavity.

**Figure 4:**
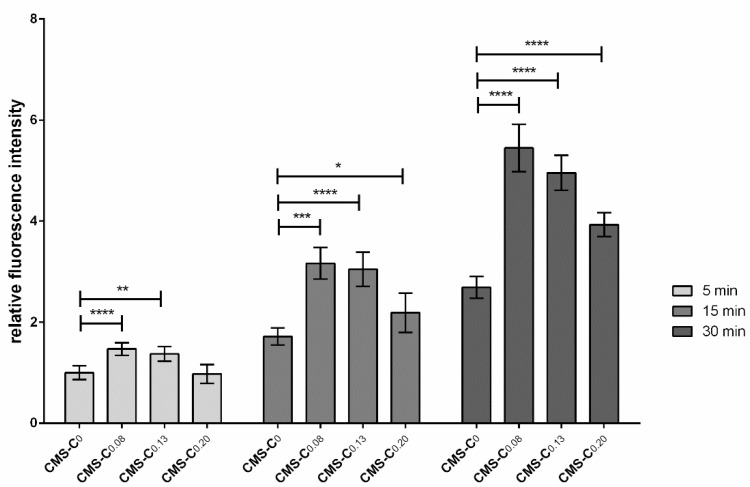
Adhesion assay under static conditions. OKG4 cells were seeded into a 96-well plate at a density of 2.5 × 10^4^ cells/well. Cell differentiation was induced by cultivating cells for 24 h in a cell culture medium containing 1.4 mM Ca^2+^. For the experiment, cells were incubated with the respective nanocarrier for 5 min, 15 min, and 30 min. After the cells were thoroughly washed with PBS, the fluorescence signals of the bound nanocarriers were determined using a GloMax Discover Microplate reader (Promega, USA). Error bars represent the standard deviation, and * indicates significant differences between the different nanocarriers. * 0.01 ≤ *p* < 0.05; ** 0.001 ≤ *p* < 0.01; *** 0.0001 ≤ *p* < 0.001; **** *p* < 0.0001.

First, adhesion of the catechol-functionalized nanocarriers was tested in a static adhesion assay at different time points. For this purpose, the immortalized gingival keratinocytes OKG4 were seeded into a 96-well plate. Epithelial cell differentiation was induced by increasing the Ca^2+^ concentration, allowing a continuous mucosal barrier to form. To determine the binding properties, the NR-loaded nanocarriers were applied to the moist monolayer for 5 min, 15 min, and 30 min (Figure 4). The monolayer was then washed thoroughly, and NR fluorescence, which indirectly represents the bound nanocarrier, was determined. Here, it was shown that the adhesion of all nanocarriers to the cell monolayer layer increased over time (Figure 4). At each time point, the catechol-functionalized nanocarriers bound more strongly to the monolayer than the non-functionalized nanocarrier CMS-C_0_. Surprisingly, an increase in catechol functionalization did not necessarily result in improved adhesion. While the adhesion of CMS-C_0.08_ and CMS-C_0.13_ was comparable at all recorded time points, the CMS-C_0.2_ adhered significantly weaker. Nonetheless, the catechol-functionalized nanocarriers exhibited improved adhesion to the mucosal model compared to the non-functionalized CMS-C_0_ after a very short time, which is an important prerequisite for topical application to the oral mucosa [1,32].

**Figure 5:**
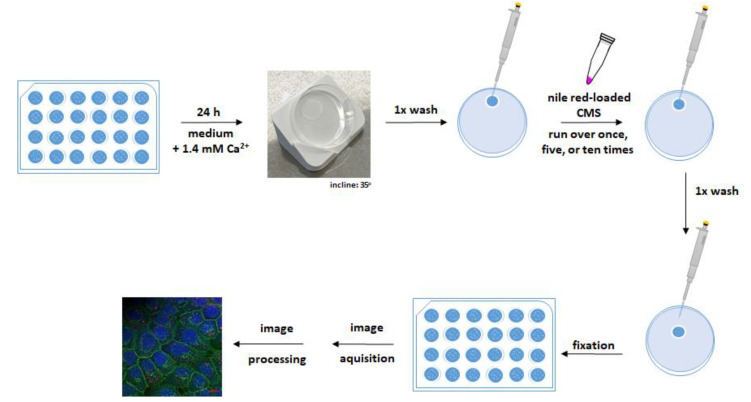
Adhesion assay under dynamic conditions. OKG4 cells were seeded on coverslips in a density of 3.5 × 10^4^ cells and placed in a 24-well plate. Cell differentiation was induced by cultivating cells for 24 h in a cell culture medium containing 1.4 mM Ca^2+^. For the experiment, the coverslip was removed from the well and placed on a petri dish inclined at 35° from the horizontal. After the cells were washed once with PBS, they were rinsed one, five, or ten times with a NR-loaded CMS nanocarrier. Finally, the cells were washed with PBS and fixed. The fixed cells were stained as described in the text, and images were acquired and processed for analysis.

However, topical therapy at the oral mucosa is further challenged by the constant salivation and exudation of crevicular fluid. To incorporate these challenges and mimic the practical application of nanocarriers, a dynamic adhesion assay was performed using OKG4 cells. Unlike the static adhesion assay, here cells were cultured and differentiated on a coverslip (Figure 5).

The NR-loaded nanocarriers were passed along the cell monolayer, which was inclined at 35 degrees, at a constant flow rate once, five, and ten times. After the nanocarrier application, the monolayer was washed to simulate the flushing effect of saliva. The bound nanocarriers were detected by confocal microscopy due to their fluorescent cargo NR.

For a better orientation, plasma membranes of fixed cells and cells’ nuclei were visualized with the Alexa Fluor 488-conjugated lectin wheat germ agglutinin (WGA) and Hoechst 33,342 (H0), respectively. Cell monolayers incubated in cell culture medium only served as negative controls (Figure S14). No NR signals were detected in the negative control indicating that the signals detected in the treated cells originated from NR and, thus, from nanocarriers attached to the cells.

**Figure 6:**
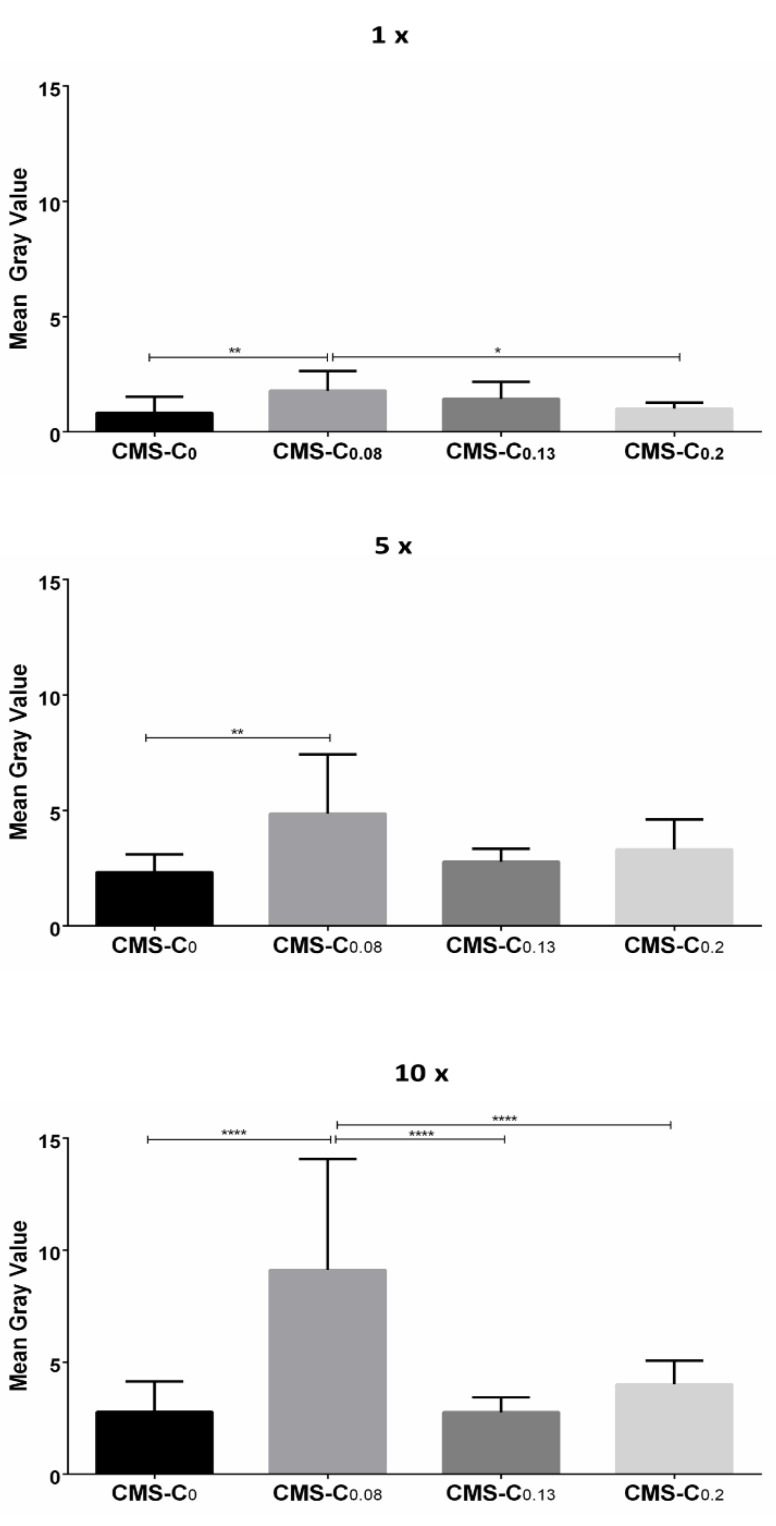
Quantification of NR fluorescence on cell monolayers after different numbers of applications (1×, 5×, and 10× rinsing). The statistical significance between the two treatments was determined using Tukey’s or Dunn’s multiple comparisons test. Mean gray value (*y*-axis) was used as a quantitative parameter. Compared to CMS-C_0_, CMS-C_0.08_ had the strongest NR signal after one, five, and ten applications. * 0.01 ≤ *p* < 0.05; ** 0.001 ≤ *p* < 0.01; **** *p* < 0.0001.

Quantification of the NR signals revealed that the fluorescence intensity increased with the frequency of application for all nanocarriers (Figure 6).

After passing along once, the nanocarriers adhered weakly to the cells, with the catechol-functionalized nanocarrier CMS-C_0.08_ exhibiting a stronger fluorescence signal than the non-functionalized CMS-C_0_. The significantly increased adhesion of the CMS-C_0.08_ compared to the non-functionalized CMS-C_0_ was also evident after applying the nanocarrier five and ten times. With respect to the higher functionalized nanocarriers, the results of the static adhesion assay were confirmed in the dynamic adhesion assay. Again, increasing the catechol content above 8% did not lead to improved adhesion_._ This effect was more pronounced in the dynamic assay than in the static assay, since here stronger forces presumably act on the carriers due to the flow and thus prevent weaker adhesion events. A possible explanation for the superior adhesion of CMS-C_0.08_ over the two CMS nanocarriers with higher catechol content may be that the higher degree of catechol functionalization increases the tendency for aggregation, and, thus, this may impair the adhesive properties.

To investigate the localization of NR-loaded nanocarrier upon application to cells further, xz projections of WGA/NR overlays of all approaches were acquired (Figure S15). The reconstructed orthogonal projections revealed the localization of the NR signal (red) and cells at the same plane indicating at least binding of the nanocarrier to the cells. In addition, the overlap of the NR signal (red) with the cell membrane (green) was evident in cells treated with CMS-C_0.08_ (Figure S15; 1× and 10×). This indicates that the NR-loaded nanocarrier was partially incorporated into the cell membrane. This projection also showed the strongest signals in cells treated with the CMS-C_0.08_ (Figure S15).

Taken together, this is proof of concept that catechol functionalization of the CMS nanocarrier leads to improved adhesion to differentiated oral mucosal cells.

## 4. Conclusions

The chemically comparable CMS nanocarriers CMS-C_0.2_, CMS-C_0.13_, and CMS-C_0.08_ were synthesized. These amphiphilic systems were functionalized with either 20%, 13%, or 8% catechol on their surfaces. All three were obtained with a size of 10 nm which is beneficial due to the mucosal mesh-pore size ranging between 10 and 200 nm [13]. By evaluating their stability, it was proven that the catechol moieties do not tend toward fast agglomeration in their unprotected state, allowing further testing on their mucoadhesive properties. To evaluate the adhesive properties of the nanocarriers, they were loaded with the fluorescent model drug NR and tested for adhesion to monolayers of differentiated gingival keratinocytes. Using two complementary adhesion assays, we showed that catechol functionalization improved the adhesive property of the CMS nanocarriers. In both assays, the CMS nanocarrier CMS-C_0.08_, with 8% catechol functionalization, exhibited the strongest binding to gingival keratinocytes and may therefore be able to deliver drugs more effectively than the other candidates. In conclusion, the new adhesive CMS nanocarrier with 8% catechol functionalization is a potential drug delivery system for the topical treatment of the oral mucosa.

Altogether, this study shows that catechol functionalization improved the adhesive properties of CMS nanocarriers, suggesting that this modification may constitute an innovative step forward for increased adhesion and penetration and thus higher drug bioavailability. Future studies should build upon our proof-of-concept by investigating in further detail the interactions between the catechol-functionalized nanocarriers and the mucosal surface, the drug release mechanism, and the possible penetration of the nanocarriers into epithelial cells.

## Figures and Tables

**Table 1:** Structural and morphological properties of the adhesive CMS nanocarriers.

CMS	M_n_, theor., calc. (kDa)	Expected DF	DF, NMR ^1^	M_n_, NMR ^1^ (kDa)	D_H_, vol ^2^ (nm)	PDI	ζ-Potential
CMS-C_0.2_	106	79.97% **2** 20% **3**	80% **2** 20% **3**	106	9.7 ± 0.5	0.95	5.2
CMS-C_0.13_	104	84.97% **2** 15% **3**	70% **2** 13% **3**	86	10.01 ± 0.08	0.73	3.8
CMS-C_0.08_	102	89.97% **2** 10% **3**	92% **2** 8% **3**	102	10.1 ± 0.6	1.04	3.1

^1^ Determined using ^1^H NMR. ^2^ Determined using DLS.

## Data Availability

Data are contained within the article or Supplementary Materials.

## Supplementary Materials

The following are available online at https://www.mdpi.com/article/10.3390/pharmaceutics14050940/s1, Figure S1: 1H NMR spectrum of 3,4-O-(1-Methylethylidene)dihydrocaffeic acid 5, Figure S2: 1H NMR spectrum of PEG-Catechol 6, Figure S3: 1H NMR spectrum of double shell 2, Figure S4: 1H NMR spectrum of double shell 3, Figure S5: 1H NMR spectrum of CMS nanocarrier CMS-C0.2, Figure S6: 1H NMR spectrum of CMS nanocarrier CMS-C0.13, Figure S7: 1H NMR spectrum of CMS nanocarrier CMS-C0.08, Figure S8: GPC spectrum of the C0.2 CMS nanocarrier, Figure S9: GPC spectrum of the C0.13 CMS nanocarrier, Figure S10: GPC spectrum of the C0.08 CMS nanocarrier, Figure S11: DLS measurements of the CMS carriers by intensity. (A) C0.08, (B) C0.15, (C) C0.2, (D) C0, Figure S12: DLS measurements of the CMS carriers by volume. (A) C0.08, (B) C0.15, (C) C0.2, (D) C0, Figure S13: Cryo-TEM image of CMS-C0.08 at 28k magnification (left) and enlarged section of the marked area (right) after 2 days of sample storage, Figure S14: Negative control of the dynamic adhesion test, Figure S15: Representative orthogonal (xz) projections of all approaches, Figure S16: Dynamic adhesion tests with 1-time rinsing, replica 1, Figure S17: Dynamic adhesion tests with 5-times rinsing, replica 1, Figure S18: Dynamic adhesion tests with 10-times rinsing, replica 1, Figure S19: Dynamic adhesion tests with 1-time rinsing, replica 2, Figure S20: Dynamic adhesion tests with 5-times rinsing, replica 2, Figure S21: Dynamic adhesion tests with 10-times rinsing, replica 2, Figure S22: Dynamic adhesion tests with 1-time rinsing, replica 3, Figure S23: Dynamic adhesion tests with 5-times rinsing, replica 3, Figure S24: Dynamic adhesion tests with 10-times rinsing, replica 3.

## References

[1] Jager J., Obst K., Lohan S.B., Viktorov J., Staufenbiel S., Renz H., Unbehauen M., Haag R., Hedtrich S., Teutloff C. (2018). Characterization of hyperbranched core-multishell nanocarriers as an innovative drug delivery system for the application at the oral mucosa. J. Periodontal Res..

[2] Demmer R.T., Papapanou P.N. (2010). Epidemiologic patterns of chronic and aggressive periodontitis. Periodontology 2000.

[3] Kassebaum N.J., Bernabé E., Dahiya M., Bhandari B., Murray C.J.L., Marcenes W. (2014). Global burden of severe periodontitis in 1990-2010: A systematic review and meta-regression. J. Dent. Res..

[4] Gonzáles J.R., Herrmann J.M., Boedeker R.H., Francz P.I., Biesalski H., Meyle J. (2001). Concentration of interleukin-1beta and neutrophil elastase activity in gingival crevicular fluid during experimental gingivitis. J. Clin. Periodontol..

[5] Hagesaether E., Adamczak M.I., Hiorth M., Tho I., Peltonen L., Peltonen L.J. (2020). Characterization of Bioadhesion, Mucin-interactions and Mucosal Permeability of Pharmaceutical Nano- and Microsystems. Characterization of Pharmaceutical Nano- and Microsystems.

[6] Woodley J. (2001). Bioadhesion: New possibilities for drug administration?. Clin. Pharmacokinet..

[7] Kim K., Kim K., Ryu J.H., Lee H. (2015). Chitosan-catechol: A polymer with long-lasting mucoadhesive properties. Biomaterials.

[8] Waite J.H. (2008). Mussel power. Nat. Mater..

[9] Appenroth J., Moreno Ostertag L., Imre A.M., Valtiner M., Mears L.L.E. (2021). Mechanistic understanding of catechols and integration into an electrochemically cross-linked mussel foot inspired adhesive hydrogel. Biointerphases.

[10] Catron N.D., Lee H., Messersmith P.B. (2006). Enhancement of poly(ethylene glycol) mucoadsorption by biomimetic end group functionalization. Biointerphases.

[11] Zhang Y., Olofsson K., Fan Y., Sánchez C.C., Andrén O.C.J., Qin L., Fortuin L., Jonsson E.M., Malkoch M. (2021). Novel Therapeutic Platform of Micelles and Nanogels from Dopa-Functionalized Triblock Copolymers. Small.

[12] Lee H., Dellatore S.M., Miller W.M., Messersmith P.B. (2007). Mussel-inspired surface chemistry for multifunctional coatings. Science.

[13] Lai S.K., O’Hanlon D.E., Harrold S., Man S.T., Wang Y.-Y., Cone R., Hanes J. (2007). Rapid transport of large polymeric nanoparticles in fresh undiluted human mucus. Proc. Natl. Acad. Sci. USA.

[14] Schimpel C., Teubl B., Absenger M., Meindl C., Fröhlich E., Leitinger G., Zimmer A., Roblegg E. (2014). Development of an advanced intestinal in vitro triple culture permeability model to study transport of nanoparticles. Mol. Pharm..

[15] Kurniasih I.N., Keilitz J., Haag R. (2015). Dendritic nanocarriers based on hyperbranched polymers. Chem. Soc. Rev..

[16] Wei Q., Achazi K., Liebe H., Schulz A., Noeske P.-L.M., Grunwald I., Haag R. (2014). Mussel-inspired dendritic polymers as universal multifunctional coatings. Angew. Chem. Int. Ed..

[17] Wei Q., Becherer T., Mutihac R.-C., Noeske P.-L.M., Paulus F., Haag R., Grunwald I. (2014). Multivalent anchoring and cross-linking of mussel-inspired antifouling surface coatings. Biomacromolecules.

[18] Yu L., Cheng C., Ran Q., Schlaich C., Noeske P.-L.M., Li W., Wei Q., Haag R. (2017). Bioinspired Universal Monolayer Coatings by Combining Concepts from Blood Protein Adsorption and Mussel Adhesion. ACS Appl. Mater. Interfaces.

[19] Schlaich C., Wei Q., Haag R. (2017). Mussel-Inspired Polyglycerol Coatings with Controlled Wettability: From Superhydrophilic to Superhydrophobic Surface Coatings. Langmuir.

[20] Li M., Gao L., Schlaich C., Zhang J., Donskyi I.S., Yu G., Li W., Tu Z., Rolff J., Schwerdtle T. (2017). Construction of Functional Coatings with Durable and Broad-Spectrum Antibacterial Potential Based on Mussel-Inspired Dendritic Polyglycerol and in Situ-Formed Copper Nanoparticles. ACS Appl. Mater. Interfaces.

[21] Schlaich C., Li M., Cheng C., Donskyi I.S., Yu L., Song G., Osorio E., Wei Q., Haag R. (2018). Mussel-Inspired Polymer-Based Universal Spray Coating for Surface Modification: Fast Fabrication of Antibacterial and Superhydrophobic Surface Coatings. Adv. Mater. Interfaces.

[22] Li M., Schlaich C., Willem Kulka M., Donskyi I.S., Schwerdtle T., Unger W.E.S., Haag R. (2019). Mussel-inspired coatings with tunable wettability, for enhanced antibacterial efficiency and reduced bacterial adhesion. J. Mater. Chem. B.

[23] Kulka M.W., Donskyi I.S., Wurzler N., Salz D., Özcan Ö., Unger W.E.S., Haag R. (2019). Mussel-Inspired Multivalent Linear Polyglycerol Coatings Outperform Monovalent Polyethylene Glycol Coatings in Antifouling Surface Properties. ACS Appl. Bio Mater..

[24] Kulka M.W., Smatty S., Hehnen F., Bierewirtz T., Silberreis K., Nie C., Kerkhoff Y., Grötzinger C., Friedrich S., Dahms L.I. (2020). The Application of Dual-Layer, Mussel-Inspired, Antifouling Polyglycerol-Based Coatings in Ventricular Assist Devices. Adv. Mater. Interfaces.

[25] Kulka M.W., Nie C., Nickl P., Kerkhoff Y., Garg A., Salz D., Radnik J., Grunwald I., Haag R. (2020). Surface-Initiated Grafting of Dendritic Polyglycerol from Mussel-Inspired Adhesion-Layers for the Creation of Cell-Repelling Coatings. Adv. Mater. Interfaces.

[26] Czuban M., Kulka M.W., Wang L., Koliszak A., Achazi K., Schlaich C., Donskyi I.S., Di Luca M., Mejia Oneto J.M., Royzen M. (2020). Titanium coating with mussel inspired polymer and bio-orthogonal chemistry enhances antimicrobial activity against Staphylococcus aureus. Mater. Sci. Eng. C Mater. Biol. Appl..

[27] Li M., Schlaich C., Zhang J., Donskyi I.S., Schwibbert K., Schreiber F., Xia Y., Radnik J., Schwerdtle T., Haag R. (2021). Mussel-inspired multifunctional coating for bacterial infection prevention and osteogenic induction. J. Mater. Sci. Technol..

[28] Park S., Kim M., Park J., Choi W., Hong J., Lee D.W., Kim B.-S. (2021). Mussel-Inspired Multiloop Polyethers for Antifouling Surfaces. Biomacromolecules.

[29] Yu K., Alzahrani A., Khoddami S., Ferreira D., Scotland K.B., Cheng J.T.J., Yazdani-Ahmadabadi H., Mei Y., Gill A., Takeuchi L.E. (2021). Self-Limiting Mussel Inspired Thin Antifouling Coating with Broad-Spectrum Resistance to Biofilm Formation to Prevent Catheter-Associated Infection in Mouse and Porcine Models. Adv. Healthc. Mater..

[30] Pornpitchanarong C., Rojanarata T., Opanasopit P., Ngawhirunpat T., Patrojanasophon P. (2020). Catechol-modified chitosan/hyaluronic acid nanoparticles as a new avenue for local delivery of doxorubicin to oral cancer cells. Colloids Surf. B Biointerfaces.

[31] Hu S., Pei X., Duan L., Zhu Z., Liu Y., Chen J., Chen T., Ji P., Wan Q., Wang J. (2021). A mussel-inspired film for adhesion to wet buccal tissue and efficient buccal drug delivery. Nat. Commun..

[32] Dommisch H., Stolte K.N., Jager J., Vogel K., Müller R., Hedtrich S., Unbehauen M., Haag R., Danker K. (2021). Characterization of an ester-based core-multishell (CMS) nanocarrier for the topical application at the oral mucosa. Clin. Oral Investig..

[33] Schwarzl R., Du F., Haag R., Netz R.R. (2017). General method for the quantification of drug loading and release kinetics of nanocarriers. Eur. J. Pharm. Biopharm..

[34] Unbehauen M.L., Fleige E., Paulus F., Schemmer B., Mecking S., Moré S.D., Haag R. (2017). Biodegradable Core–Multishell Nanocarriers: Influence of Inner Shell Structure on the Encapsulation Behavior of Dexamethasone and Tacrolimus. Polymers.

[35] Roller S., Zhou H., Haag R. (2005). High-loading polyglycerol supported reagents for Mitsunobu- and acylation-reactions and other useful polyglycerol derivatives. Mol. Divers..

[36] Dickson M.A., Hahn W.C., Ino Y., Ronfard V., Wu J.Y., Weinberg R.A., Louis D.N., Li F.P., Rheinwald J.G. (2000). Human keratinocytes that express hTERT and also bypass a p16(INK4a)-enforced mechanism that limits life span become immortal yet retain normal growth and differentiation characteristics. Mol. Cell. Biol..

[37] Buskermolen J.K., Reijnders C.M.A., Spiekstra S.W., Steinberg T., Kleverlaan C.J., Feilzer A.J., Bakker A.D., Gibbs S. (2016). Development of a Full-Thickness Human Gingiva Equivalent Constructed from Immortalized Keratinocytes and Fibroblasts. Tissue Eng. Part C Methods.

[38] Wang Y.-Y., Lai S.K., Suk J.S., Pace A., Cone R., Hanes J. (2008). Addressing the PEG mucoadhesivity paradox to engineer nanoparticles that “slip” through the human mucus barrier. Angew. Chem. Int. Ed. Engl..

[39] Reboredo C., González-Navarro C.J., Martínez-Oharriz C., Martínez-López A.L., Irache J.M. (2021). Preparation and evaluation of PEG-coated zein nanoparticles for oral drug delivery purposes. Int. J. Pharm..

[40] Wang H., Wang L., Zhang S., Zhang W., Li J., Han Y. (2021). Mussel-inspired polymer materials derived from nonphytogenic and phytogenic catechol derivatives and their applications. Polym. Int..

[41] Li Y., Cheng J., Delparastan P., Wang H., Sigg S.J., DeFrates K.G., Cao Y., Messersmith P.B. (2020). Molecular design principles of Lysine-DOPA wet adhesion. Nat. Commun..

[42] Rajes K., Walker K.A., Hadam S., Zabihi F., Rancan F., Vogt A., Haag R. (2020). Redox-Responsive Nanocarrier for Controlled Release of Drugs in Inflammatory Skin Diseases. Pharmaceutics.

[43] Rajes K., Walker K.A., Hadam S., Zabihi F., Ibrahim-Bacha J., Germer G., Patoka P., Wassermann B., Rancan F., Rühl E. (2021). Oxidation-Sensitive Core-Multishell Nanocarriers for the Controlled Delivery of Hydrophobic Drugs. ACS Biomater. Sci. Eng..

[44] Zadik Y., Elad S., Shapira A., Shapira M.Y. (2017). Treatment of oral mucosal manifestations of chronic graft-versus-host disease: Dexamethasone vs. budesonide. Expert Opin. Pharmacother..

[45] Ryu J.H., Hong S., Lee H. (2015). Bio-inspired adhesive catechol-conjugated chitosan for biomedical applications: A mini review. Acta Biomater..

