# Supplementary material for: Novel Adhesive Nanocarriers Based on Mussel-Inspired Polyglycerols for the Application onto Mucosal Tissues
Source: Pharmaceutics. 2022 Apr 26;14(5):940. doi: 10.3390/pharmaceutics14050940 (PMC9144514; doi:10.3390/pharmaceutics14050940)
Supplement: Supplementary file 1 [file pharmaceutics-14-00940-s001.zip › pharmaceutics-1654685-supplementary.pdf]

pharmaceutics

MDPI

Article

Novel Adhesive Nanocarriers based on Mussel-Inspired Polyglycerols for the Application onto Mucosal Tissues

Keerthana Rajes [1], Peer Nölte [1], Cynthia V. Yapto [2], Kerstin Danker [2], Henrik Dommisch [2] and Rainer Haag [1,*]

1. NMR spectra

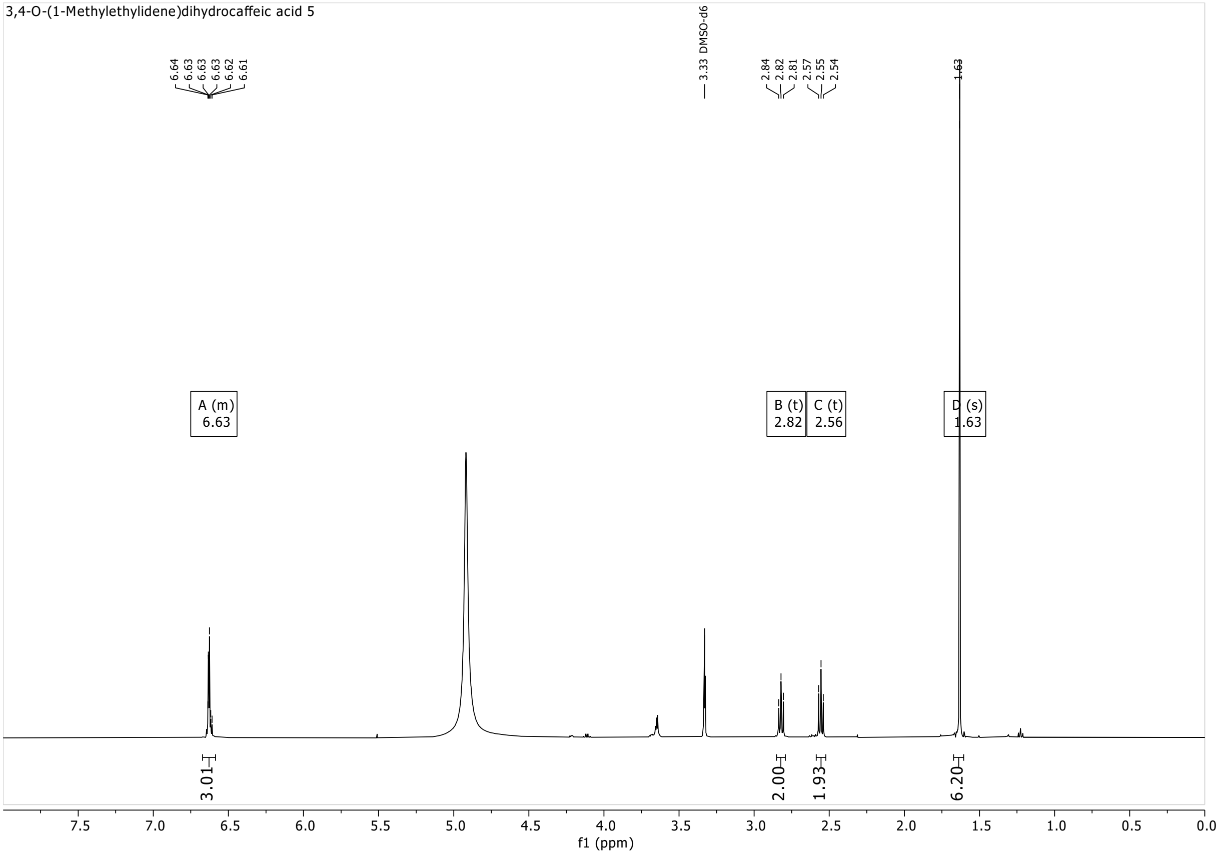

Figure S1. ^{1}H NMR spectrum of 3,4-*O*-(1-Methylethylidene)dihydrocaffeic acid **5**.

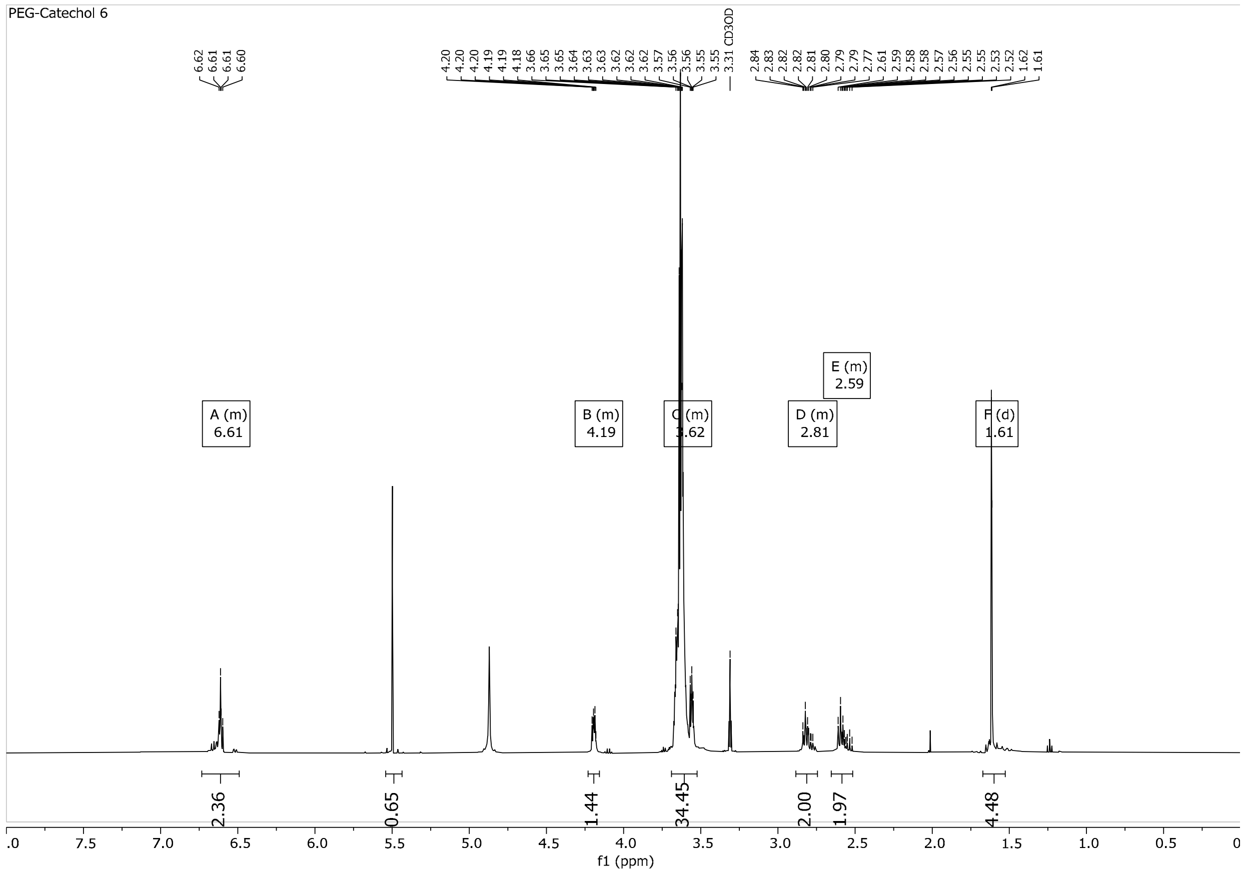

Figure S2. ^{1}H NMR spectrum of PEG-Catechol **6**.

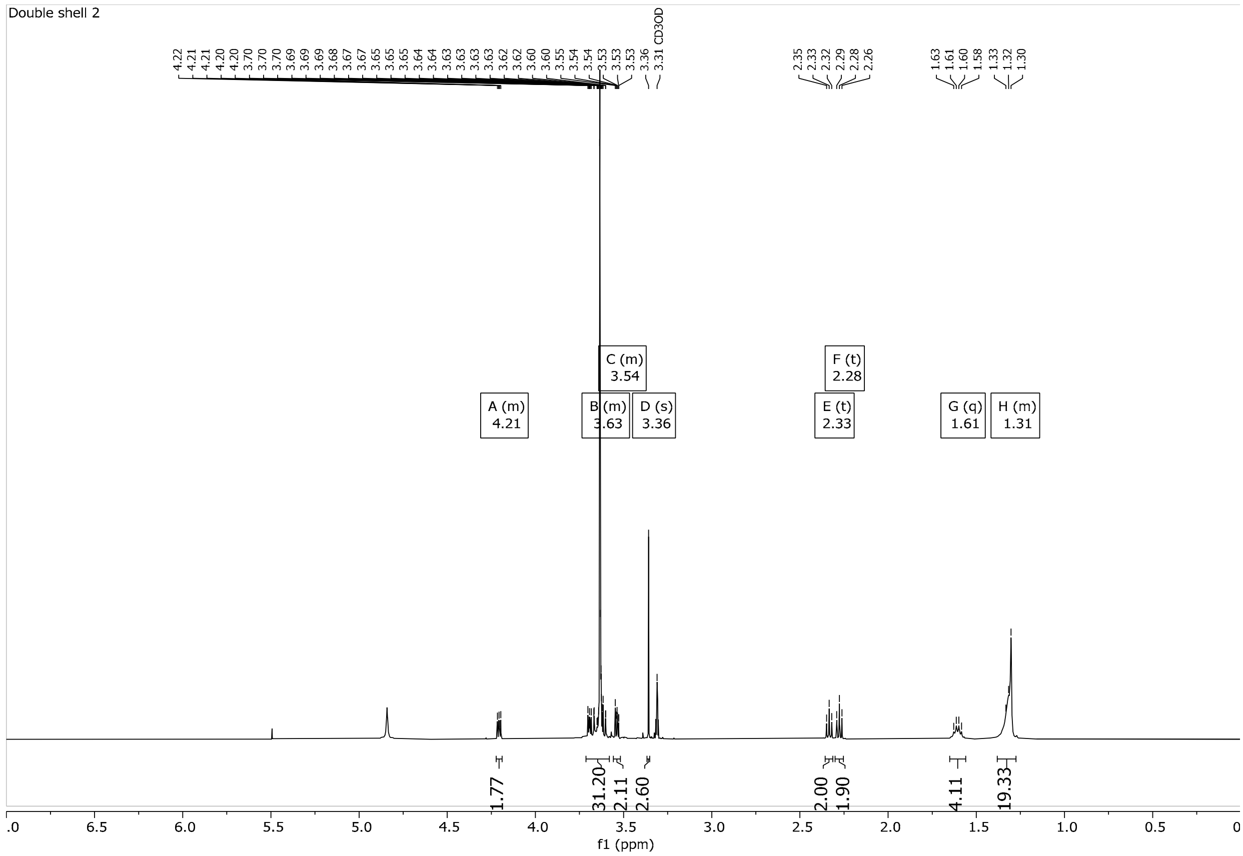

Figure S3. ^{1}H NMR spectrum of double shell **2**.

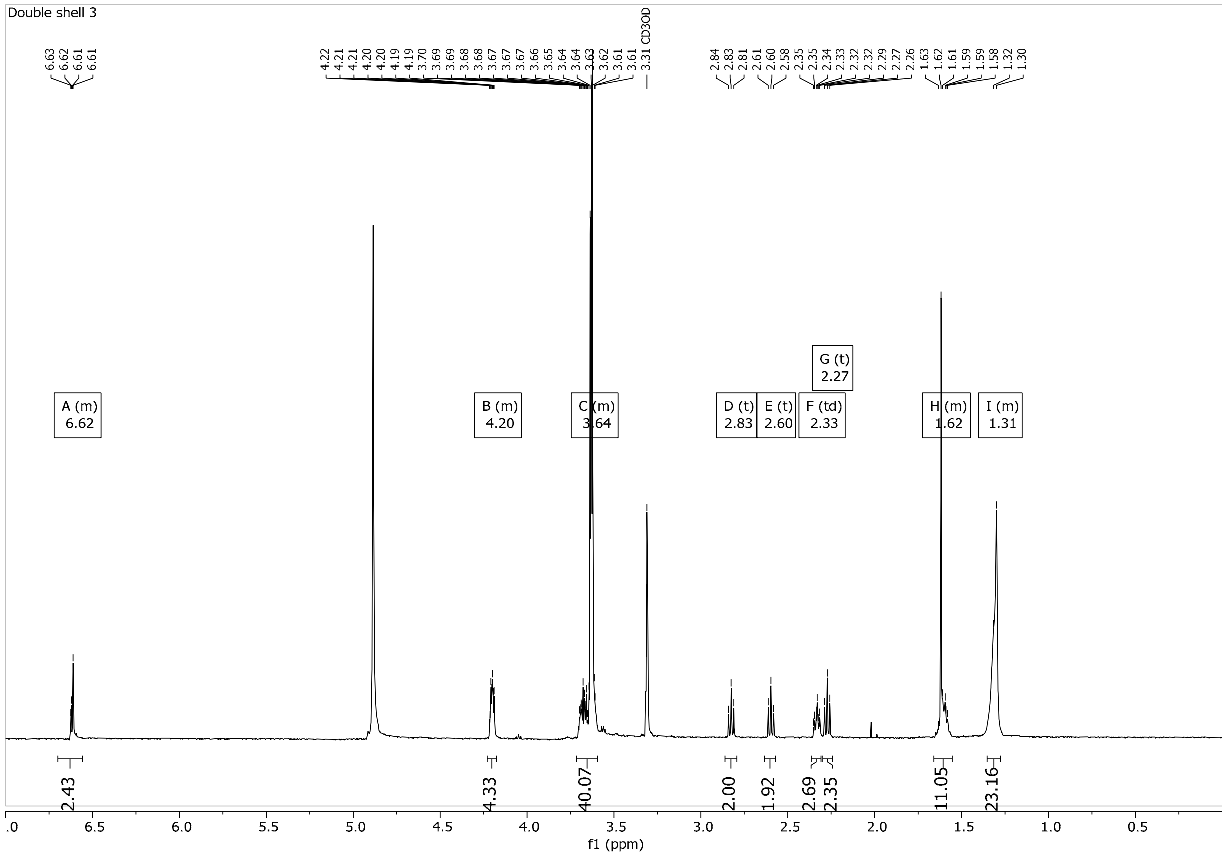

Figure S4. ^{1}H NMR spectrum of double shell **3**.

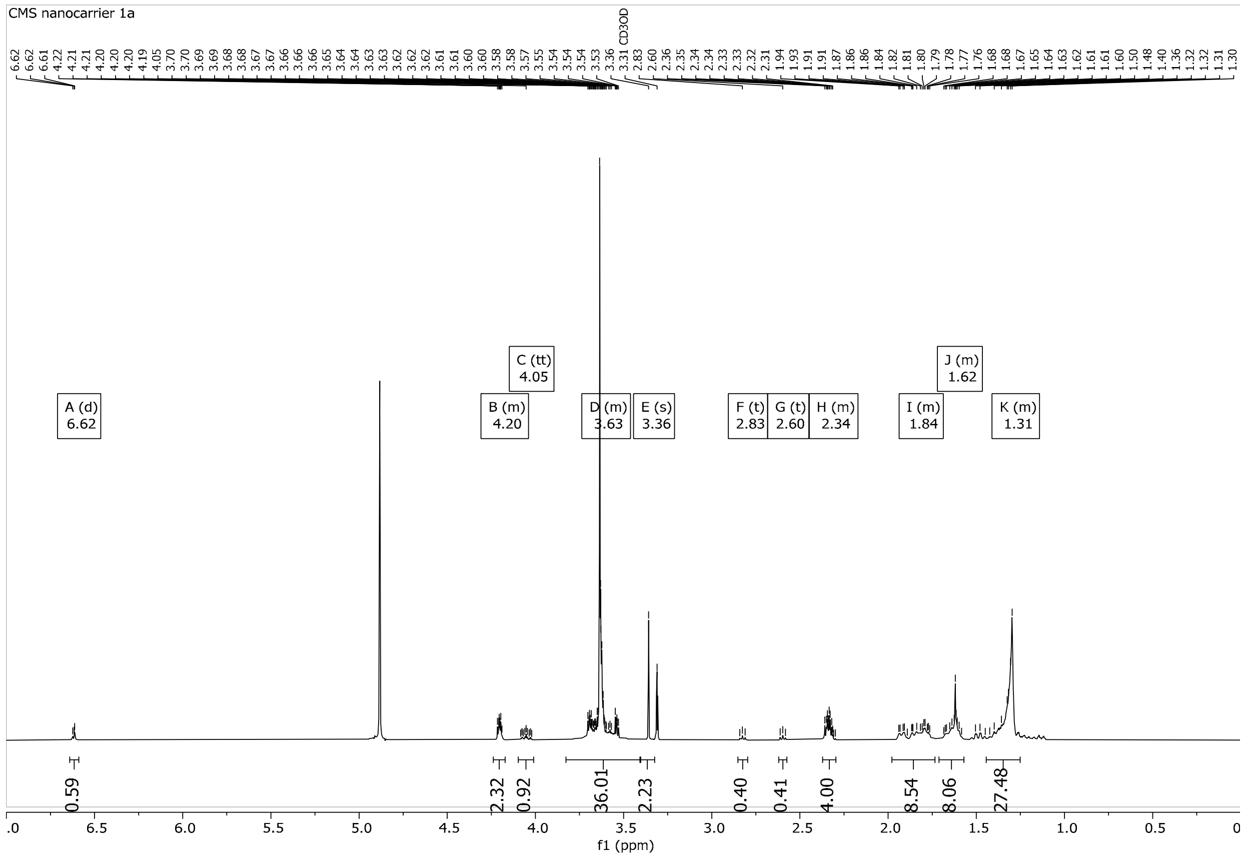

Figure S5. ^{1}H NMR spectrum of CMS nanocarrier CMS-$C_{0.2}$.

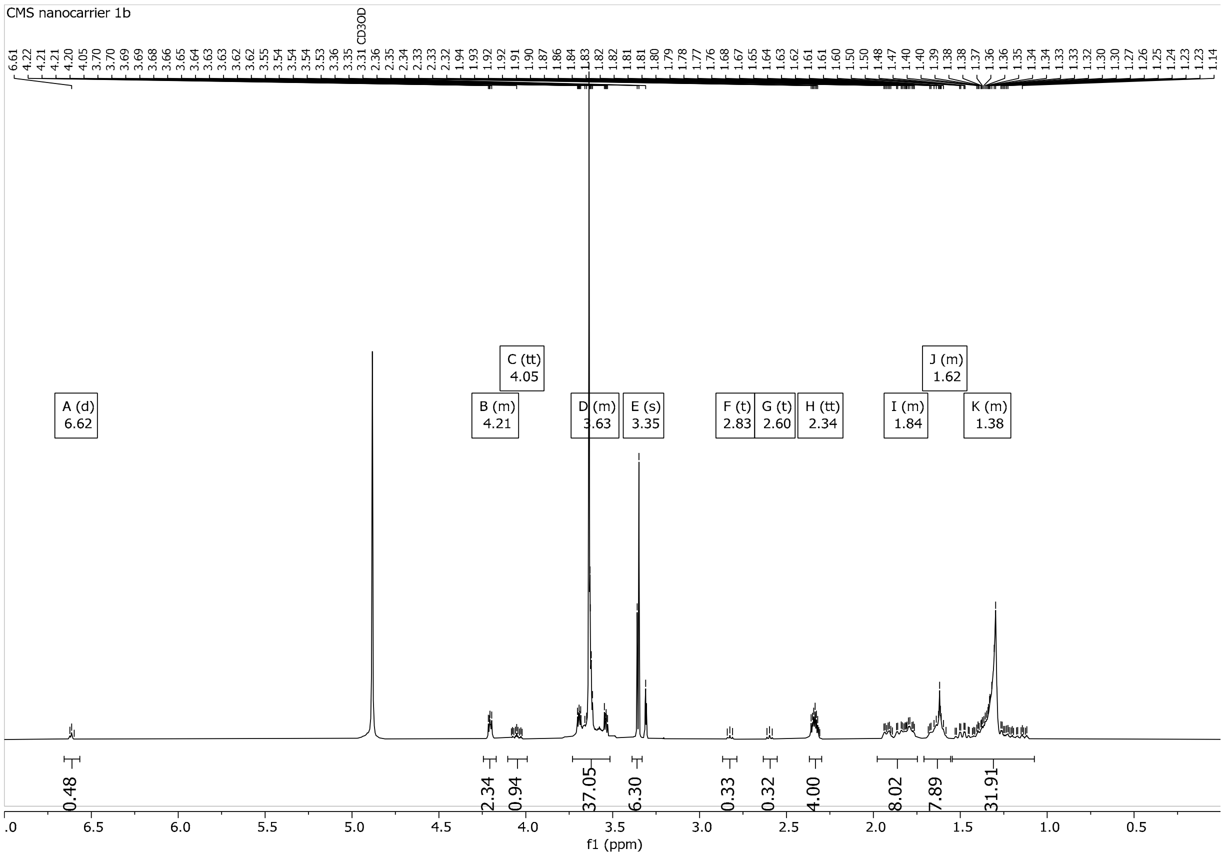

Figure S6. ^{1}H NMR spectrum of CMS nanocarrier CMS-$C_{0.13}$.

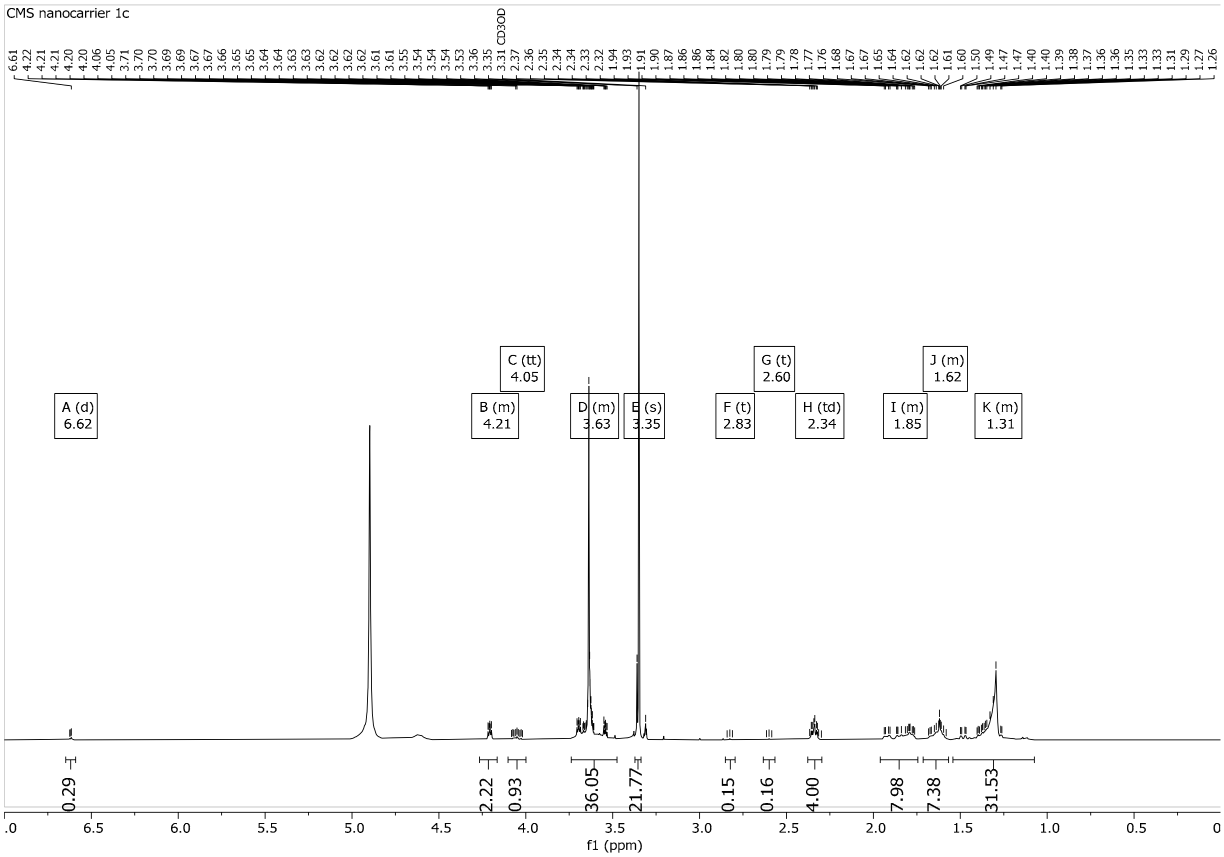

Figure S7. ^{1}H NMR spectrum of CMS nanocarrier CMS-$C_{0.08}$.

2. GPC spectra

GPC spectra showed a molecular weight M_n of 28 kDa in case of the $C_{0.2}$ CMS nanocarrier, 29 kDa in case of the $C_{0.2}$ CMS nanocarrier, and 28 kDa in case of the $C_{0.2}$ CMS nanocarrier.

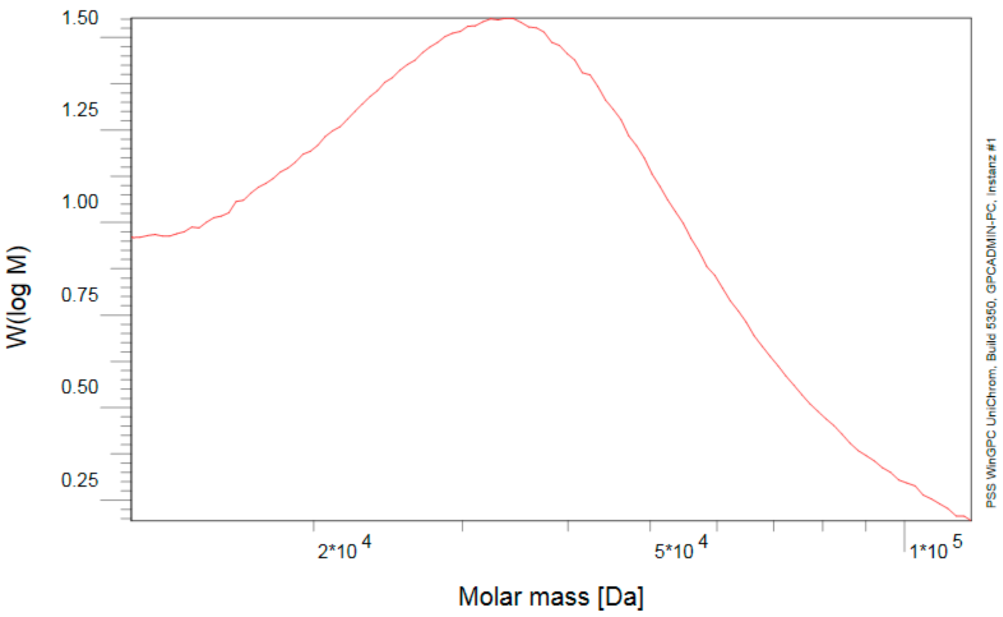

Figure S8. GPC spectrum of the $C_{0.2}$ CMS nanocarrier.

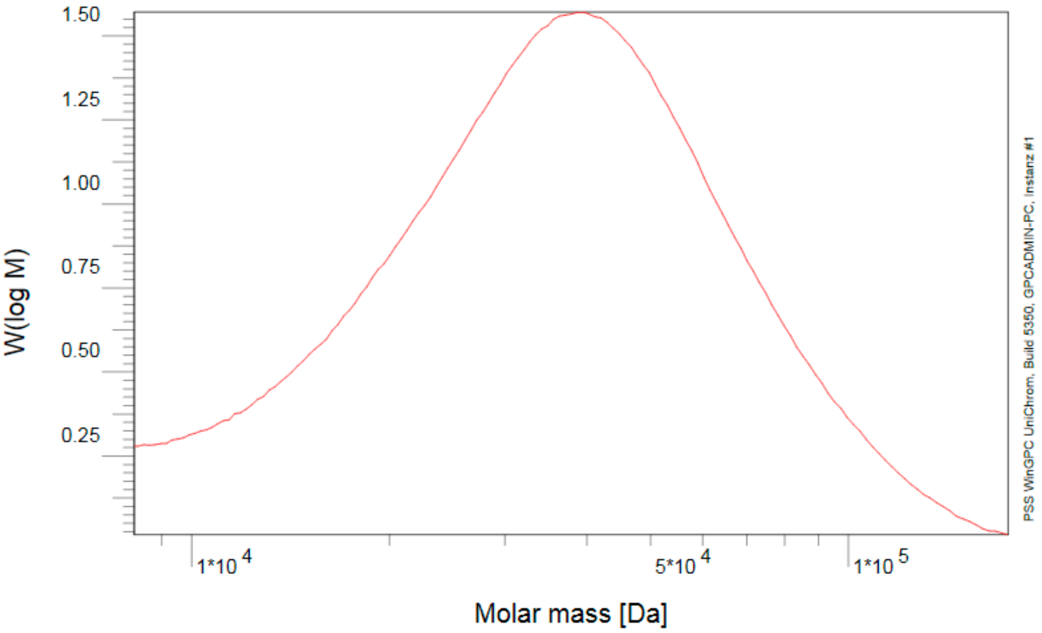

Figure S9. GPC spectrum of the $C_{0.13}$ CMS nanocarrier.

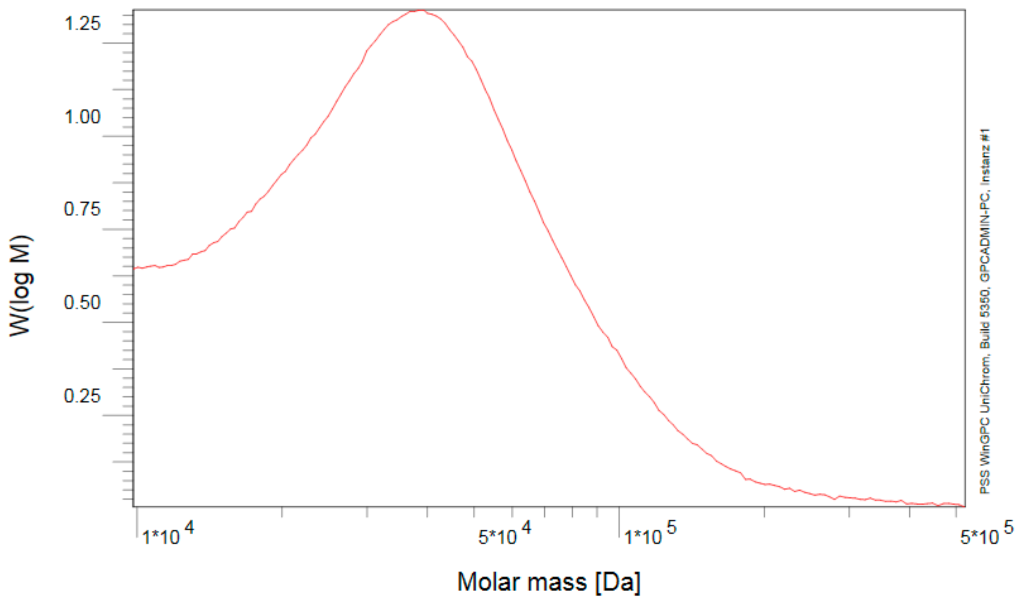

Figure S10. GPC spectrum of the $C_{0.08}$ CMS nanocarrier.

3. DLS measurements

Figure S11 depicts the hydrodynamic diameter of the carriers by intensity and figure S12 by volume. The DLS measurements by intensity also show aggregation when looking at the protected carrier system. This is a characteristic of CMS carriers and is also reported in literature. However, aggregations when looking at the intensity are multiplied and do not display the actual ratio. These are taken into consideration when looking at the volume ratio and therefore display the actual ratio better. The slight changes in the shape of the DLS curves display this. Figure S11 shows the DLS measurements of the catechol-functionalized CMS carriers (Fig. 1, A-C) as well as that of the C_0 carrier (D). All do show tendencies towards aggregation which happens regardless of the catechol functionalization. The DLS measurements by volume of all carriers are shown in figure S12 with no notable aggregation when looking at the volume ratio.

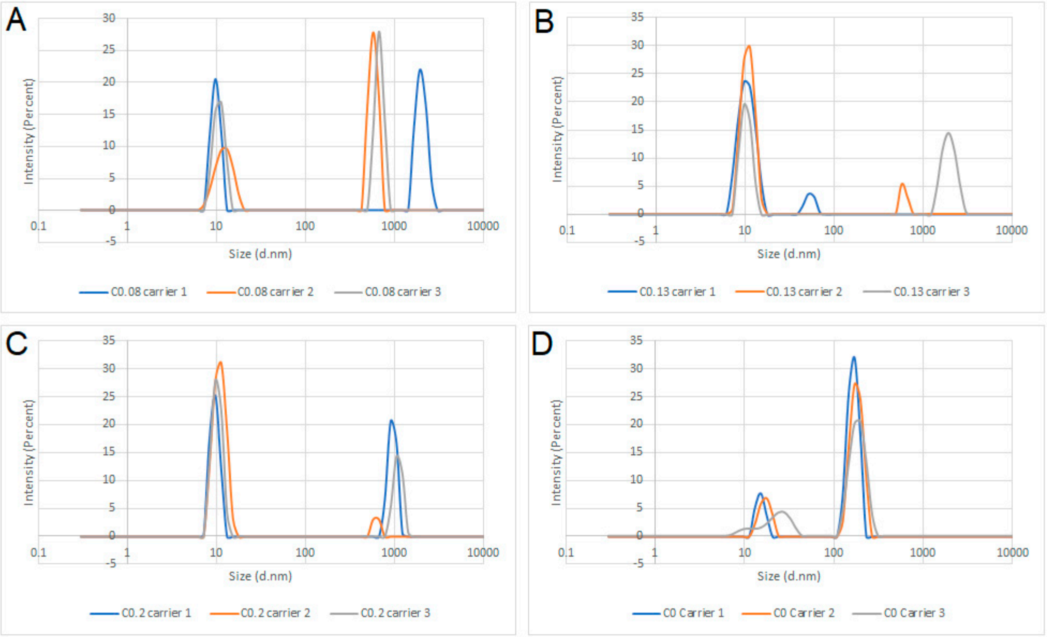

Figure S11. DLS measurements of the CMS carriers by intensity. A) $C_{0.08}$, B) $C_{0.13}$, C) $C_{0.2}$, D) C_0.

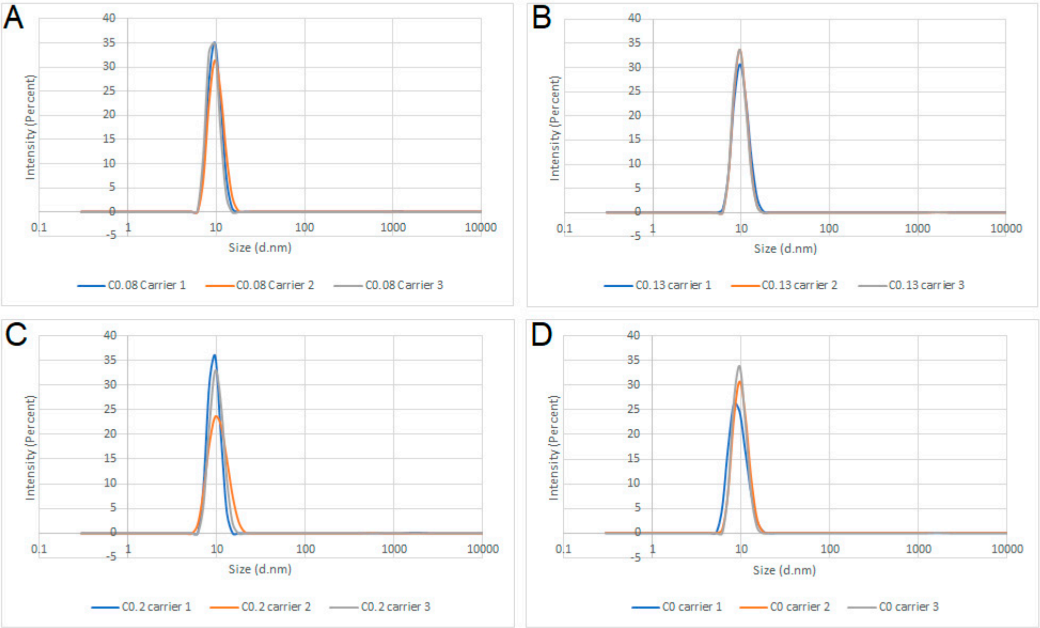

Figure S12. DLS measurements of the CMS carriers by volume. A) $C_{0.08}$, B) C0.15, C) $C_{0.2}$, D) C_{0}.

4. Cryo-TEM measurement

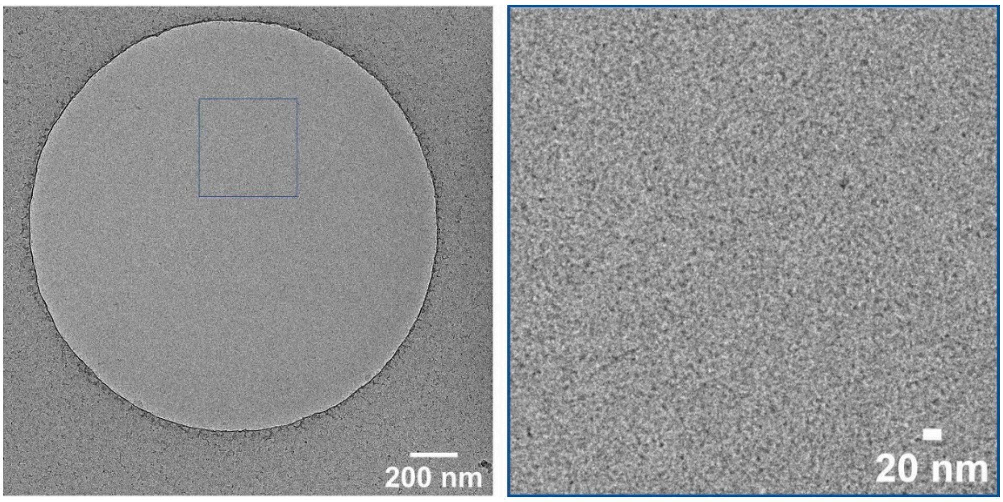

Figure S13. Cryo-TEM image of CMS-$C_{0.08}$ at 28k magnification (left) and enlarged section of the marked area (right) after 2 days of sample storage.

5. Additional data on the adhesion tests

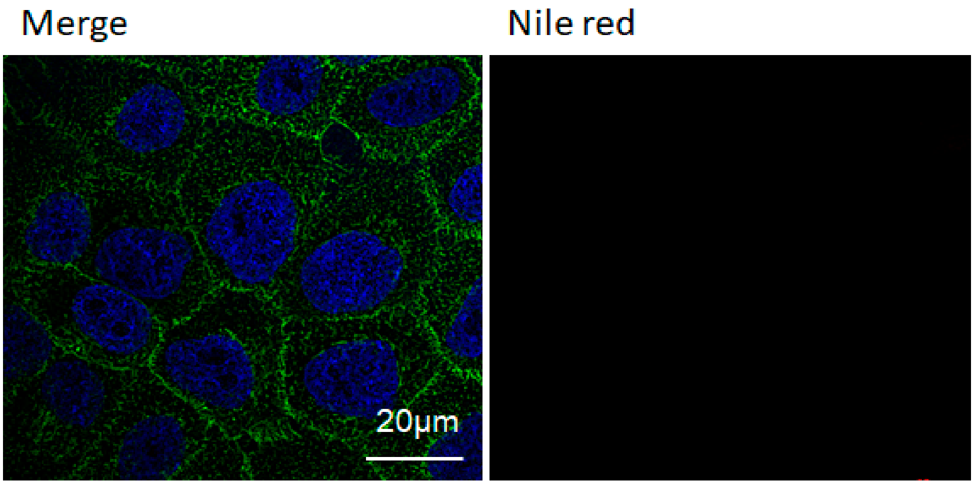

Figure S14. Negative control of the dynamic adhesion test. Representative image of OKG4 cells after 1 h incubation with cell culture medium as negative control. Cells were fixed and stained. Cell membranes (green) were visualized using Alexa-Fluor 488-conjugated wheat germ agglutinin, while cell nuclei (e) were stained with Hoechst 33342 dye. The left image shows the overlay, and the right image shows the potential autofluorescence in the red channel. Gain: 700; magnification: 63x; bar =20 µm.

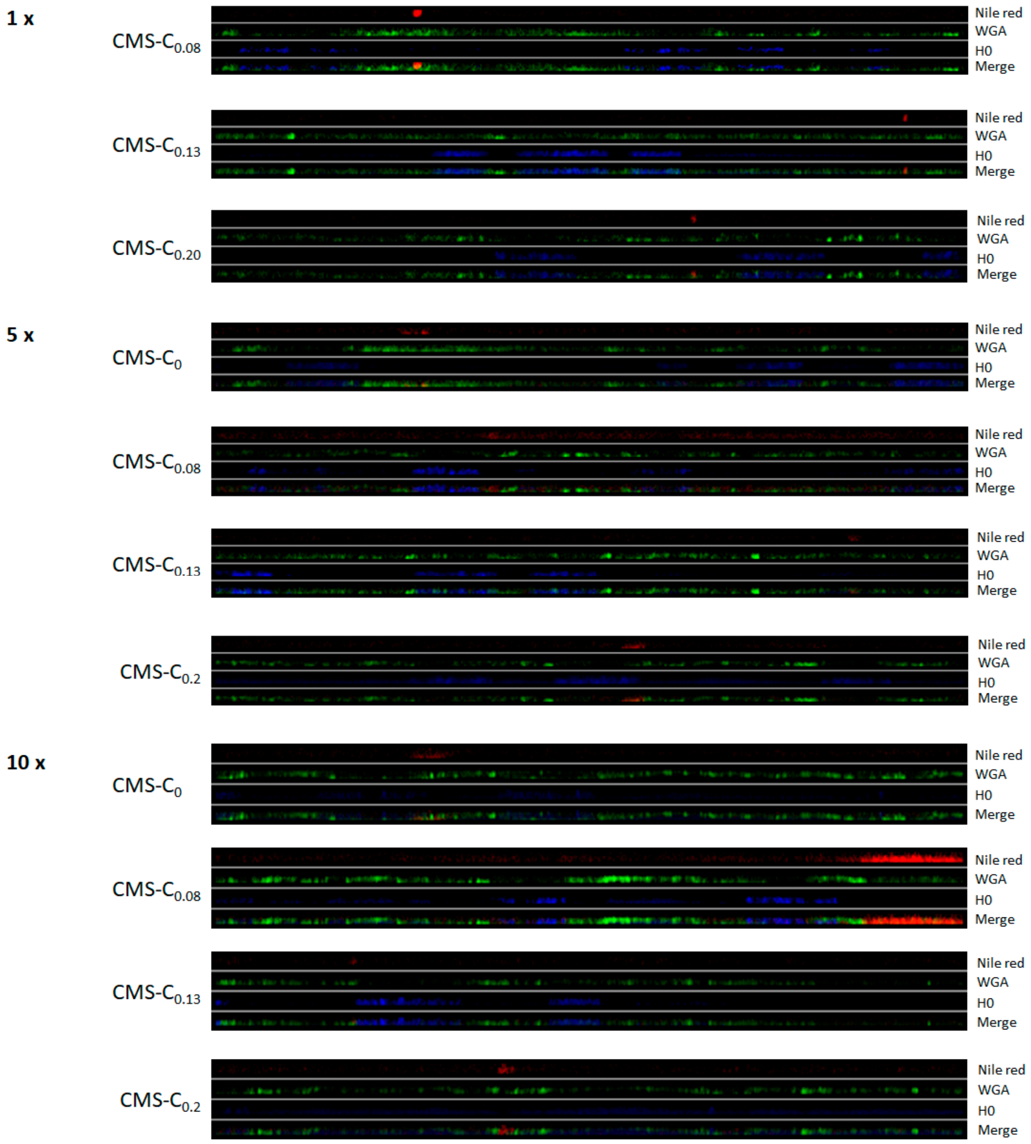

Figure S15. Representative orthogonal (xz) projections of all approaches. The xz projections showed that the NR signal (red) and the signal from the WGA-stained cell membranes (green) were localized in the same plane, indicating an interaction of the nanocarriers with the cells.

Figures S16 - S24 show all images from the three independent experiments of the dynamic adhesion test in which NR was captured. As described in the Materials and Methods section, the mean grey value of four different areas of each image was determined using ImageJ. The mean value of these four ranges was calculated and the background value was subtracted. The quantification of all signals is shown in Figure 4D.

<u>**Experiment 1**</u>

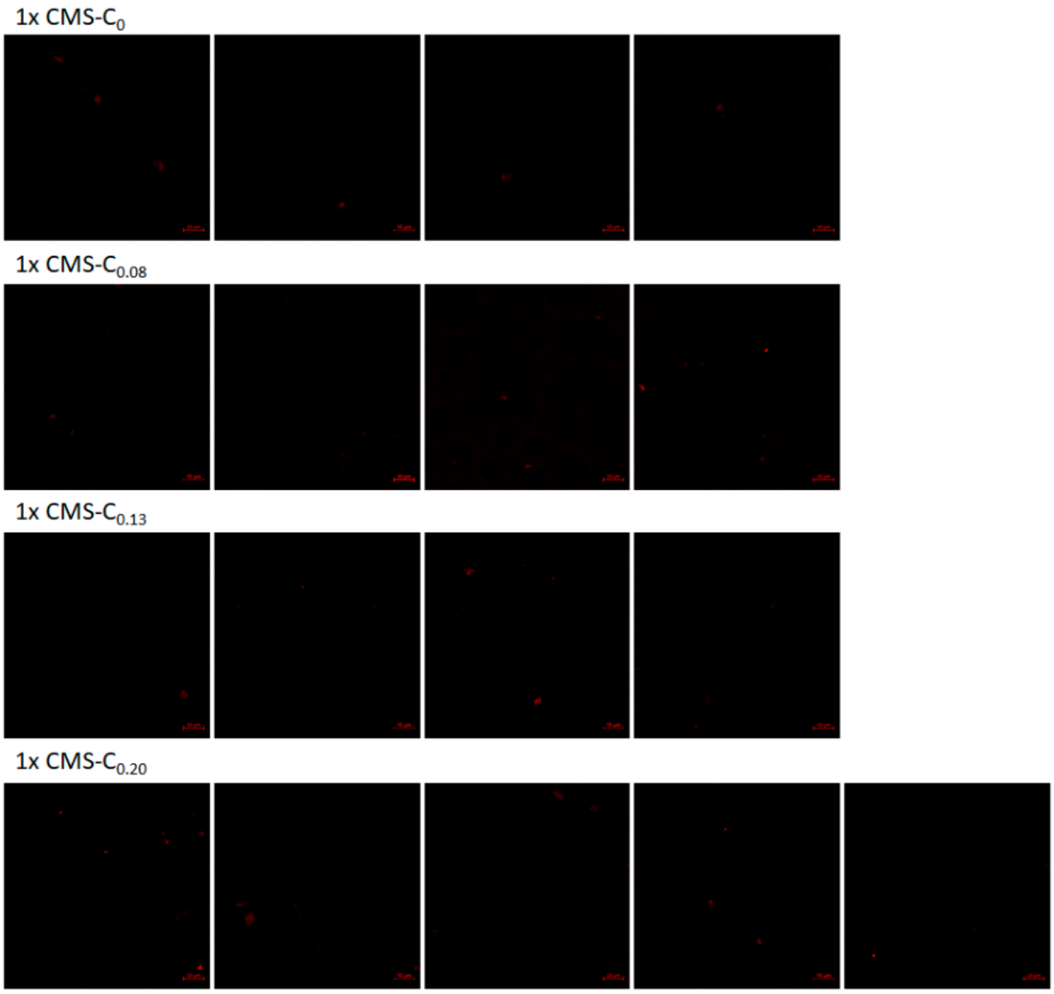

Figure S16. Dynamic adhesion tests with 1-time rinsing, replica 1. Images taken after rinsing 1 time with the nanocarriers as indicated in this figure.

<u>**Experiment 1**</u>

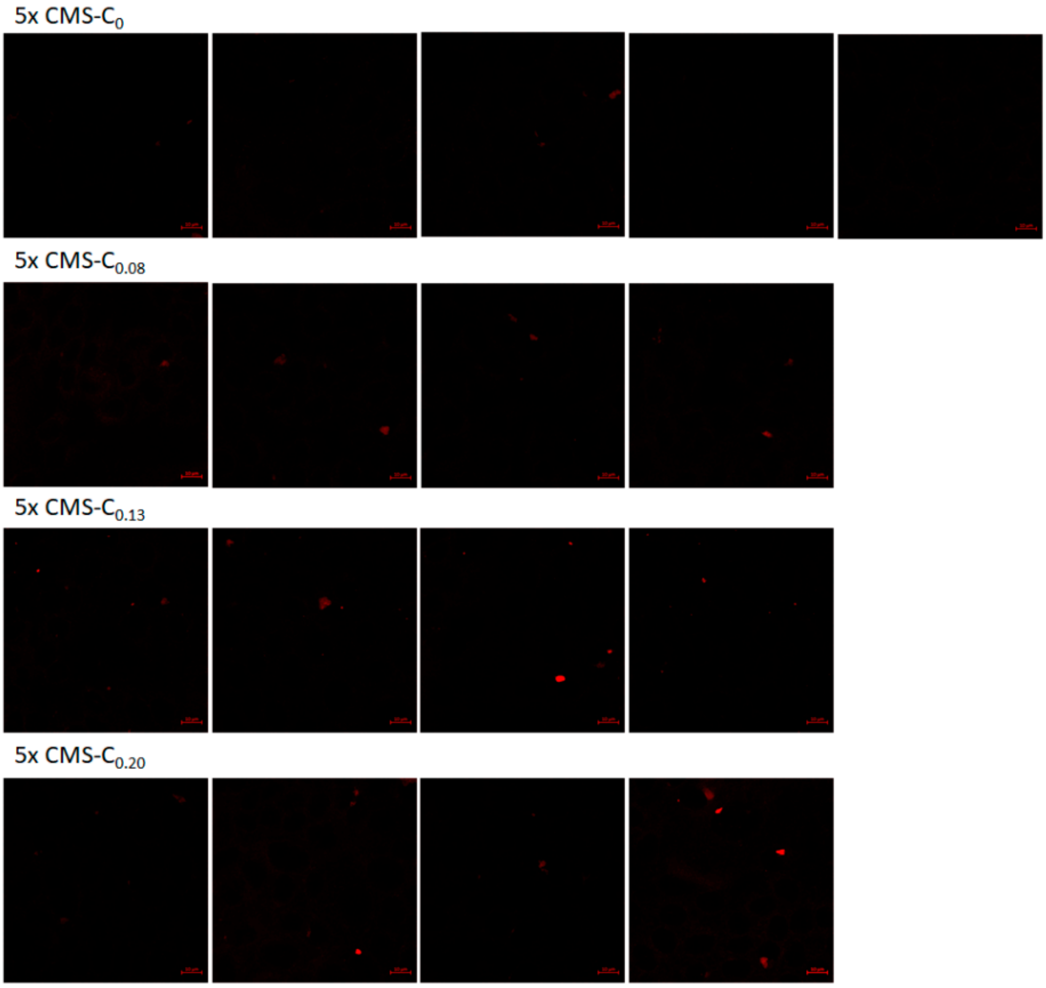

Figure S17. Dynamic adhesion tests with 5-times rinsing, replica 1. Images taken after rinsing 5 times with the nanocarriers as indicated in this figure.

Experiment 1

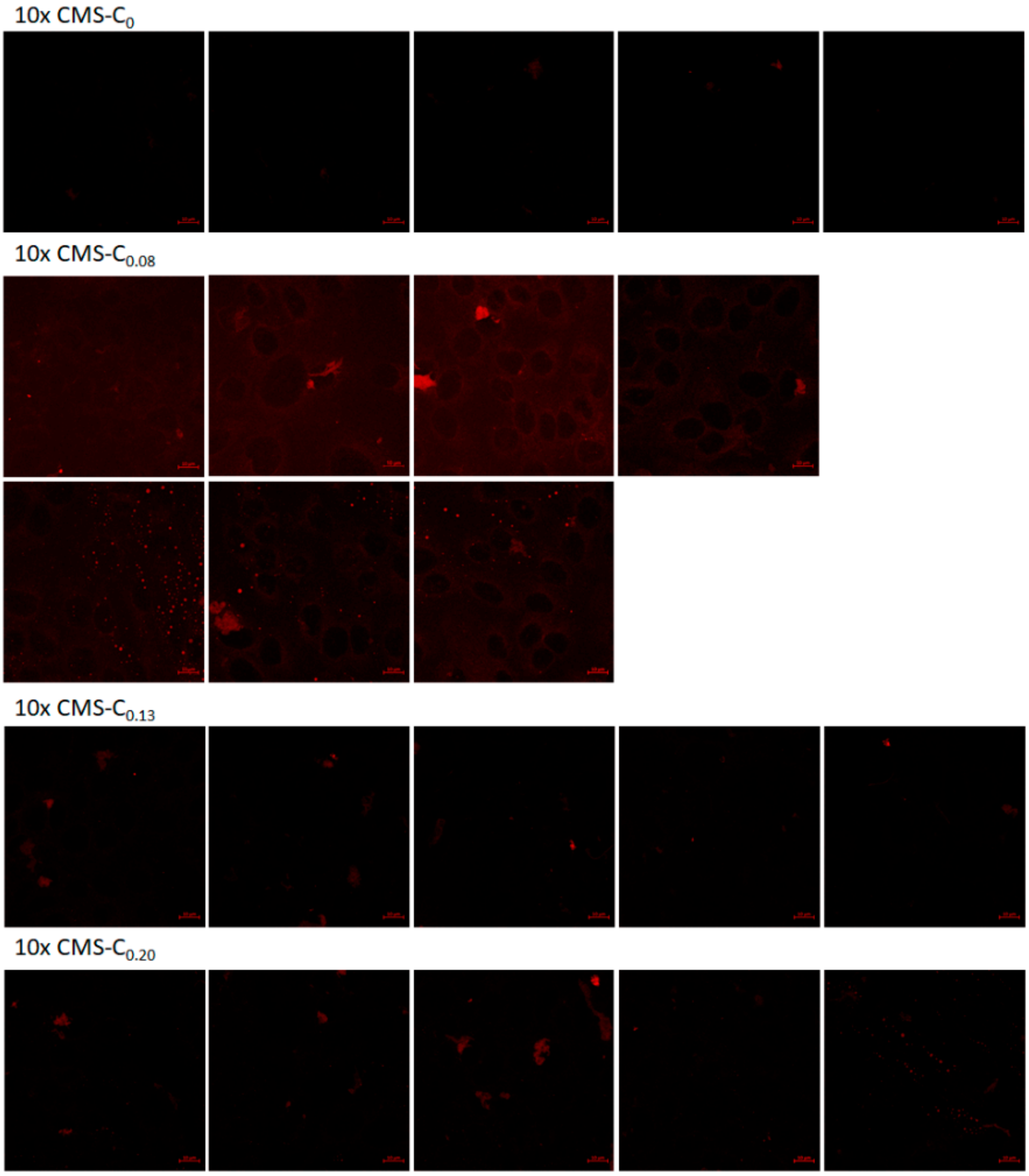

Figure S18. Dynamic adhesion tests with 10-times rinsing, replica 1. Images taken after rinsing 10 times with the nanocarriers as indicated in this figure.

Experiment 2

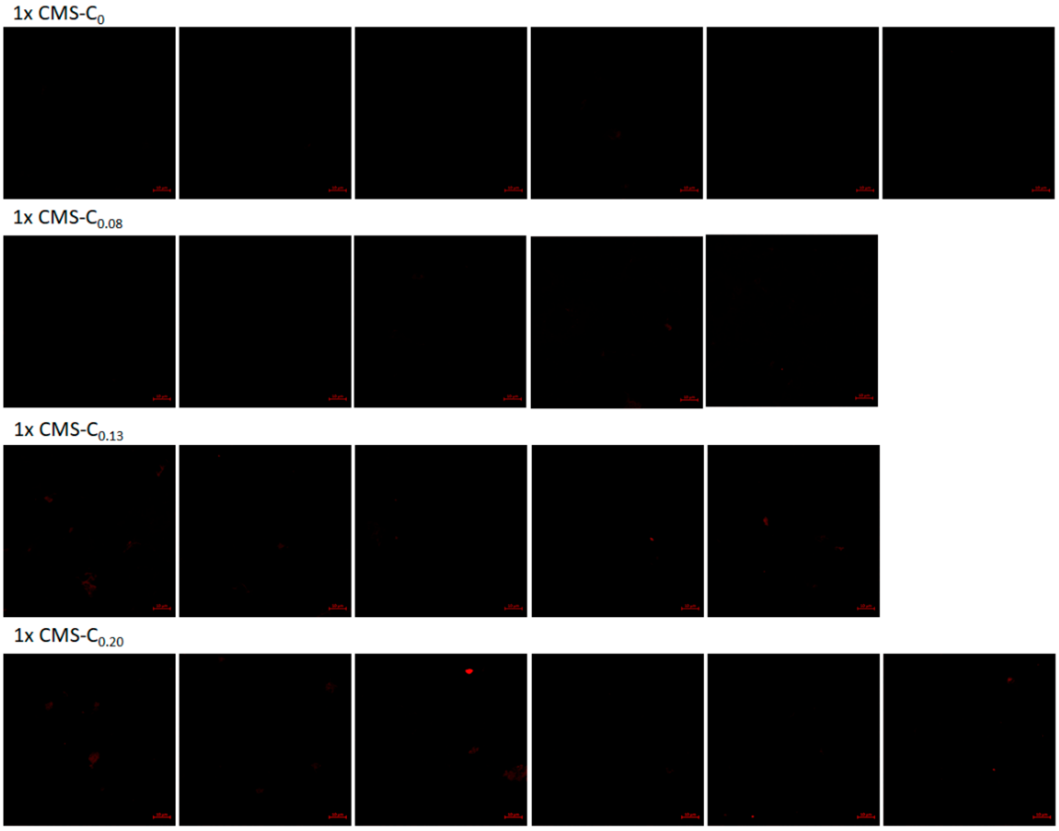

Figure S19. Dynamic adhesion tests with 1-time rinsing, replica 2. Images taken after rinsing 1 time with the nanocarriers as indicated in this figure.

Experiment 2

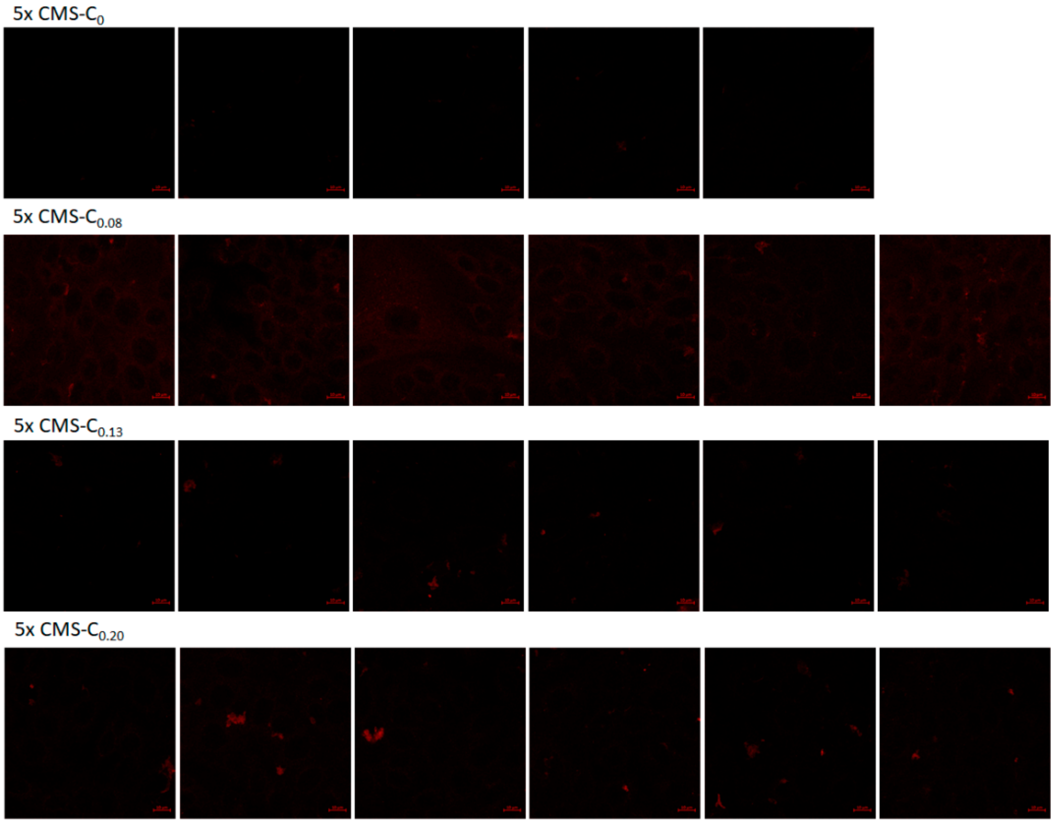

Figure S20. Dynamic adhesion tests with 5-times rinsing, replica 2. Images taken after rinsing 5 times with the nanocarriers as indicated in this figure.

Experiment 2

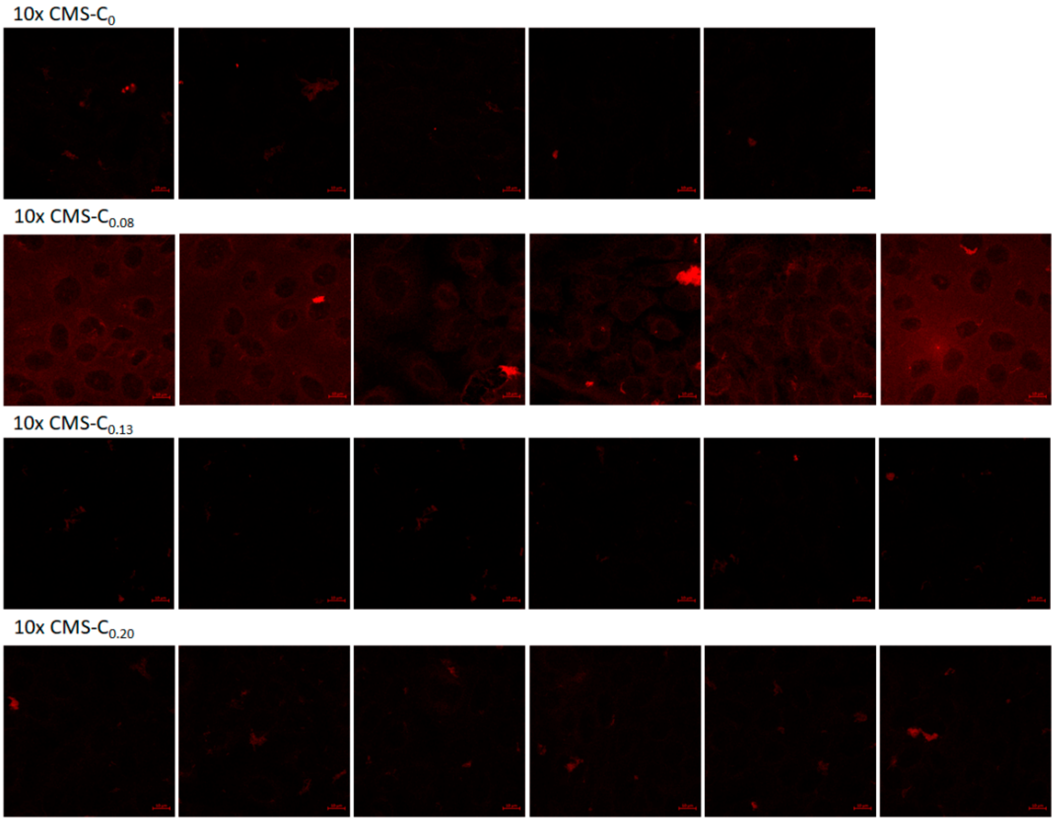

Figure S21. Dynamic adhesion tests with 10-times rinsing, replica 2. Images taken after rinsing 10 times with the nanocarriers as indicated in this figure.

Experiment 3

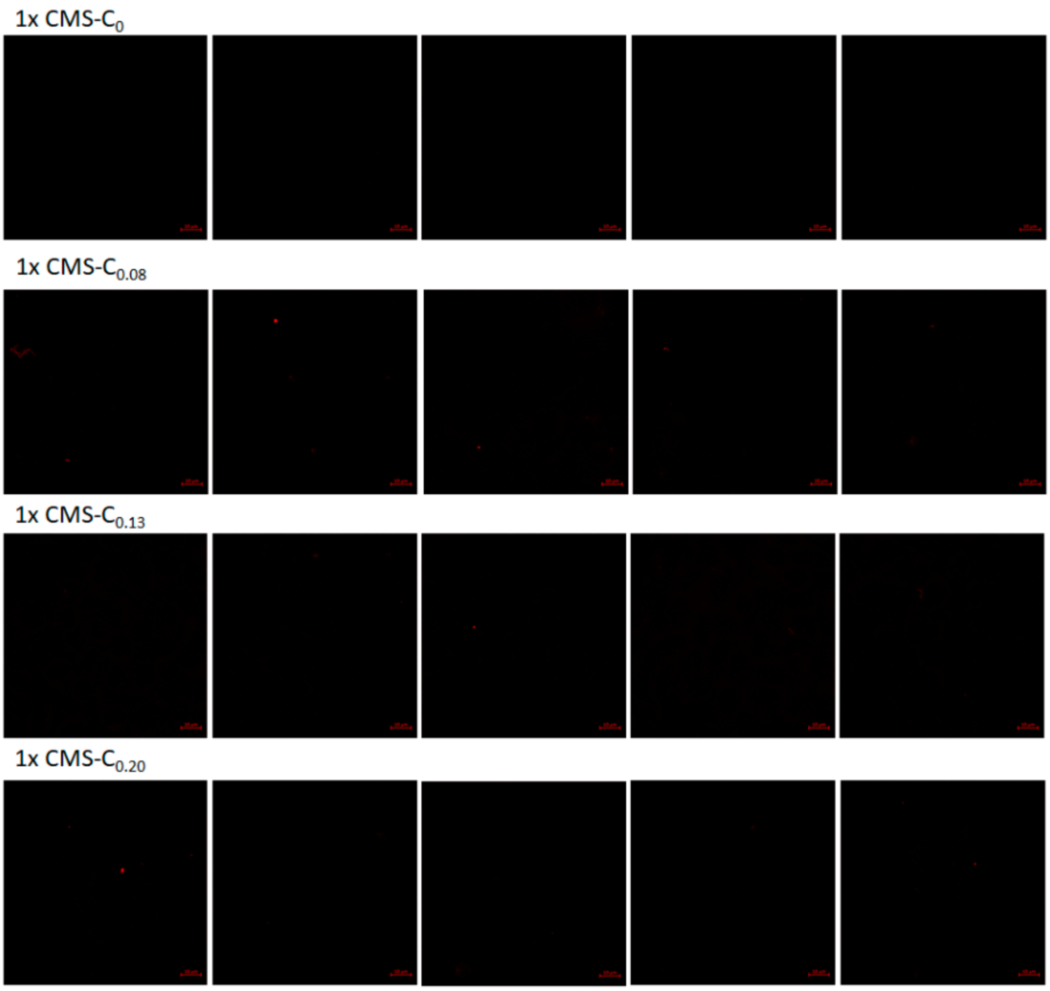

Figure S22. Dynamic adhesion tests with 1-time rinsing, replica 3. Images taken after rinsing 1 time with the nanocarriers as indicated in this figure.

Experiment 3

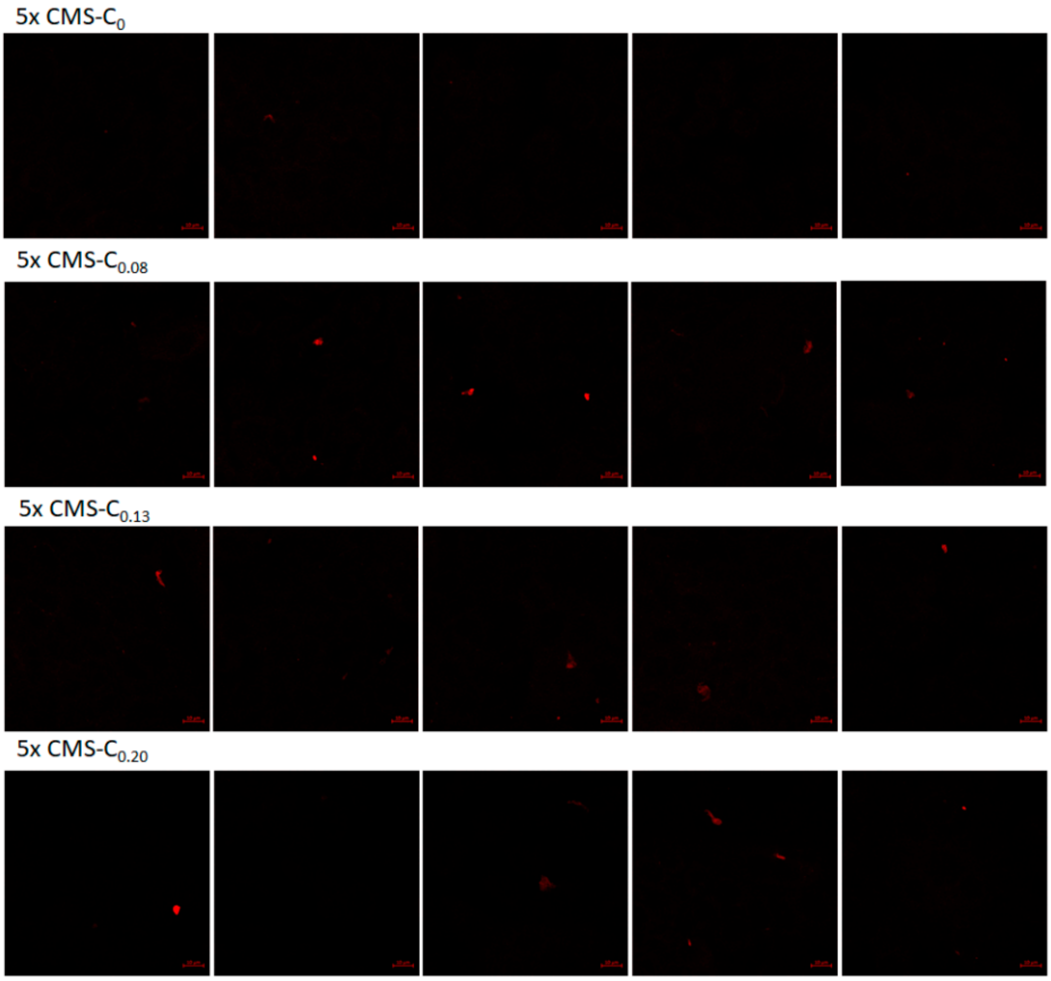

Figure S23. Dynamic adhesion tests with 5-times rinsing, replica 3. Images taken after rinsing 5 times with the nanocarriers as indicated in this figure.

Experiment 3

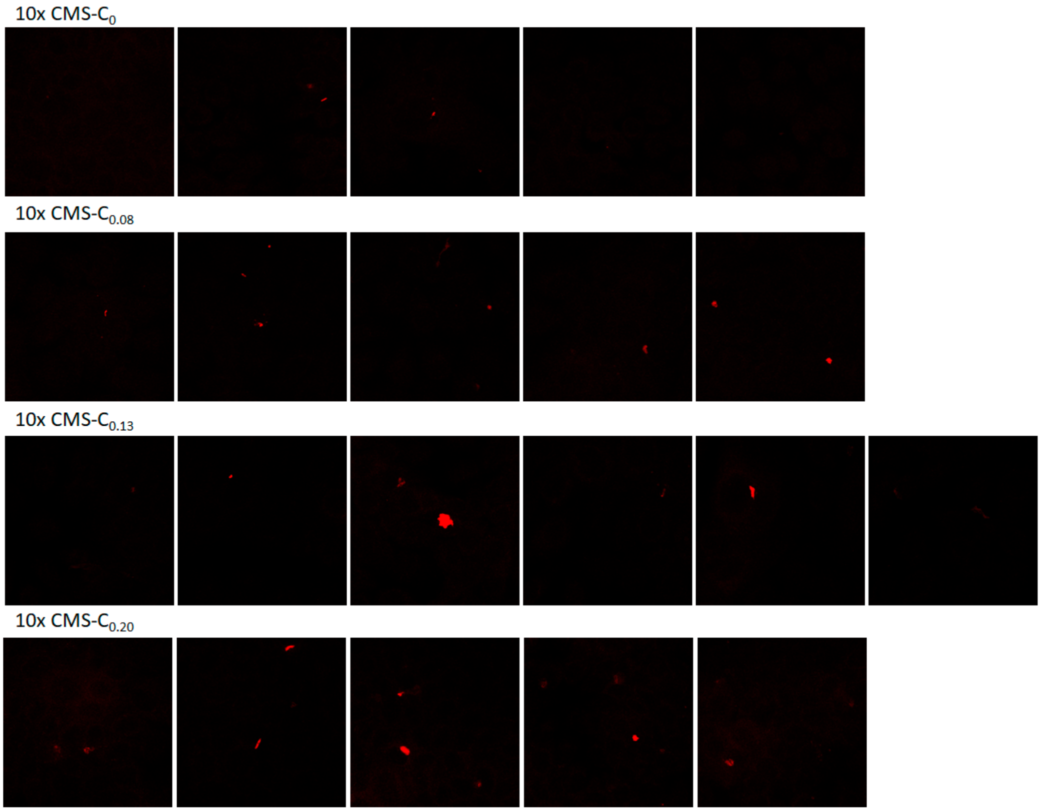

Figure S24. Dynamic adhesion tests with 10-times rinsing, replica 3. Images taken after rinsing 10 times with the nanocarriers as indicated in this figure.

6. Determination of the degree of functionalization (DF)

The molar mass was calculated by ^1H NMR spectroscopy based on the degree of functionalization DF by determining the ratio of the double shell conjugation to the hPG scaffold. Depending on the DF, the signal at 3.6 originates from 5 protons of the hPG backbone and 31 protons of the PEG backbone. With a molecular weight of 400 Da, the PEG has 9 repeating units resulting in a total amount of 36 protons. 2 protons are shifted to the downfield (4.2 ppm) while another three result from the methyl end group (3.4 ppm) and are therefore subtracted leading to 31 protons. The signal at 2.3 ppm originating from the alkyl chain of the double shell is also DF dependent. Considering both dependencies of the signal on the DF, the DF can be calculated as followed:

$$\sigma_{3.7} = 5 + 36 \cdot DF - 2 \cdot DF - 3 \cdot DF, \quad (S1)$$

$$\sigma_{3.7} = 5 + 31 \cdot DF, \quad (S2)$$

$$\sigma_{2.34} = 4 \cdot DF, \quad (S3)$$

$$\Sigma_{3.7} = \sigma_{3.7} / \sigma_{2.34} \cdot 4 = (5 + 31 \cdot DF) / DF, \quad (S4)$$

$$DF = 5 / (\Sigma_{3.7} - 31) \quad (S5)$$

Setting the signal at 2.3 ppm to 4, the total DF for the CMS nanocarriers can be calculated as followed:

$$DF(CMS\text{-}C_{0.2}) = 5 / (36 - 31) = 100\%, \quad (S6)$$

$$DF(CMS\text{-}C_{0.13}) = 5 / (37 - 31) = 83\%, \quad (S7)$$

$$DF(CMS\text{-}C_{0.08}) = 5 / (36 - 31) = 100\%, \quad (S8)$$

The DF of each double shell can be determined by looking at the ratio of the catechol peak found in double shell **2** at 2.8 ppm to the peak of the alkyl chain found in both double shells at 2.3 ppm under consideration of the total DF.
